# Integrative analysis of single-cell and bulk RNA sequencing unveils a machine learning-based pan-cancer major histocompatibility complex-related signature for predicting immunotherapy efficacy

**DOI:** 10.1007/s00262-024-03714-5

**Published:** 2024-05-07

**Authors:** Jia-Lin Feng, Bo Liang, Wen-Jie Zheng, Le Xu, Qin-Yi Zhou, Jun Chen

**Affiliations:** 1 Department of Head and Neck Surgery, Ren Ji Hospital, Shanghai Jiao Tong University School of Medicine, Shanghai, China; 2 Department of Nephrology, The Key Laboratory for the Prevention and Treatment of Chronic Kidney Disease of Chongqing, Chongqing Clinical Research Center of Kidney and Urology Diseases, Xinqiao Hospital, Army Medical University (Third Military Medical University), Chongqing, China

**Keywords:** Immunotherapy, Single‐cell RNA sequencing, Pan‐cancer, Major histocompatibility complex, Immunotherapy response, Signature

## Abstract

Major histocompatibility complex (MHC) could serve as a potential biomarker for tumor immunotherapy, however, it is not yet known whether MHC could distinguish potential beneficiaries. Single‐cell RNA sequencing datasets derived from patients with immunotherapy were collected to elucidate the association between MHC and immunotherapy response. A novel MHCsig was developed and validated using large‐scale pan‐cancer data, including The Cancer Genome Atlas and immunotherapy cohorts. The therapeutic value of MHCsig was further explored using 17 CRISPR/Cas9 datasets. MHC-related genes were associated with drug resistance and MHCsig was significantly and positively associated with immunotherapy response and total mutational burden. Remarkably, MHCsig significantly enriched 6% top‐ranked genes, which were potential therapeutic targets. Moreover, we generated Hub-MHCsig, which was associated with survival and disease-special survival of pan-cancer, especially low-grade glioma. This result was also confirmed in cell lines and in our own clinical cohort. Later low-grade glioma-related Hub-MHCsig was established and the regulatory network was constructed. We provided conclusive clinical evidence regarding the association between MHCsig and immunotherapy response. We developed MHCsig, which could effectively predict the benefits of immunotherapy for multiple tumors. Further exploration of MHCsig revealed some potential therapeutic targets and regulatory networks.

**Supplementary Information:**

The online version contains supplementary material available at 10.1007/s00262-024-03714-5.

## Introduction

Immunotherapy has been approved by the Food and Drug Administration to treat multiple cancer types and has brought significant survival benefits to patients. Neoadjuvant nivolumab plus chemotherapy resulted in significantly longer event-free survival in patients with resectable non-small-cell lung cancer [1], dostarlimab plus carboplatin-paclitaxel significantly increased progression-free survival among patients with primary advanced or recurrent endometrial cancer [2], nivolumab plus ipilimumab or nivolumab showed improved clinical outcomes in patients with advanced melanoma [3]. However, despite the success of immunotherapy, resistance to these agents restricts the number of patients able to achieve durable responses [4]. Thus, a better understanding of biomarkers is essential for optimizing patient selection and combination strategies to cope with immune resistance.

The major histocompatibility complex (MHC) is the most important region in the vertebrate genome with respect to infection and autoimmunity and is crucial in adaptive and innate immunity [5]. Immune targeting of tumor-specific antigens is a powerful therapeutic strategy. Immunotherapy targeting MHC expands the range of antigens and enables intracellular oncoproteins to directly target cell surfaces [6]. Primary response to anti-CTLA-4 requires robust melanoma MHC class I expression and primary response to anti-PD-1 is associated with preexisting IFN-γ-mediated immune activation that includes tumor-specific MHC class II expression and components of innate immunity when MHC class I is compromised [7]. Loss of MHC class I antigen presentation on tumor cells plays a critical role in the reduction of T cell infiltration during drug resistance [8]. MHC class I-mediated antigen presentation by Hodgkin Reed-Sternberg cells is an important component of the biological response to standard chemo/radiotherapy [9] and genetically driven PD-L1 expression and MHC class II positivity on Hodgkin Reed-Sternberg cells are potential predictors of favorable outcomes after PD-1 blockade [10]. In addition, higher MHC class I and MHC class II scores significantly enriched in melanoma of unknown primary compared with known primary [11]. All of the evidence suggests that MHC could serve as a potential biomarker for tumor immunotherapy.

**Fig. 1:**
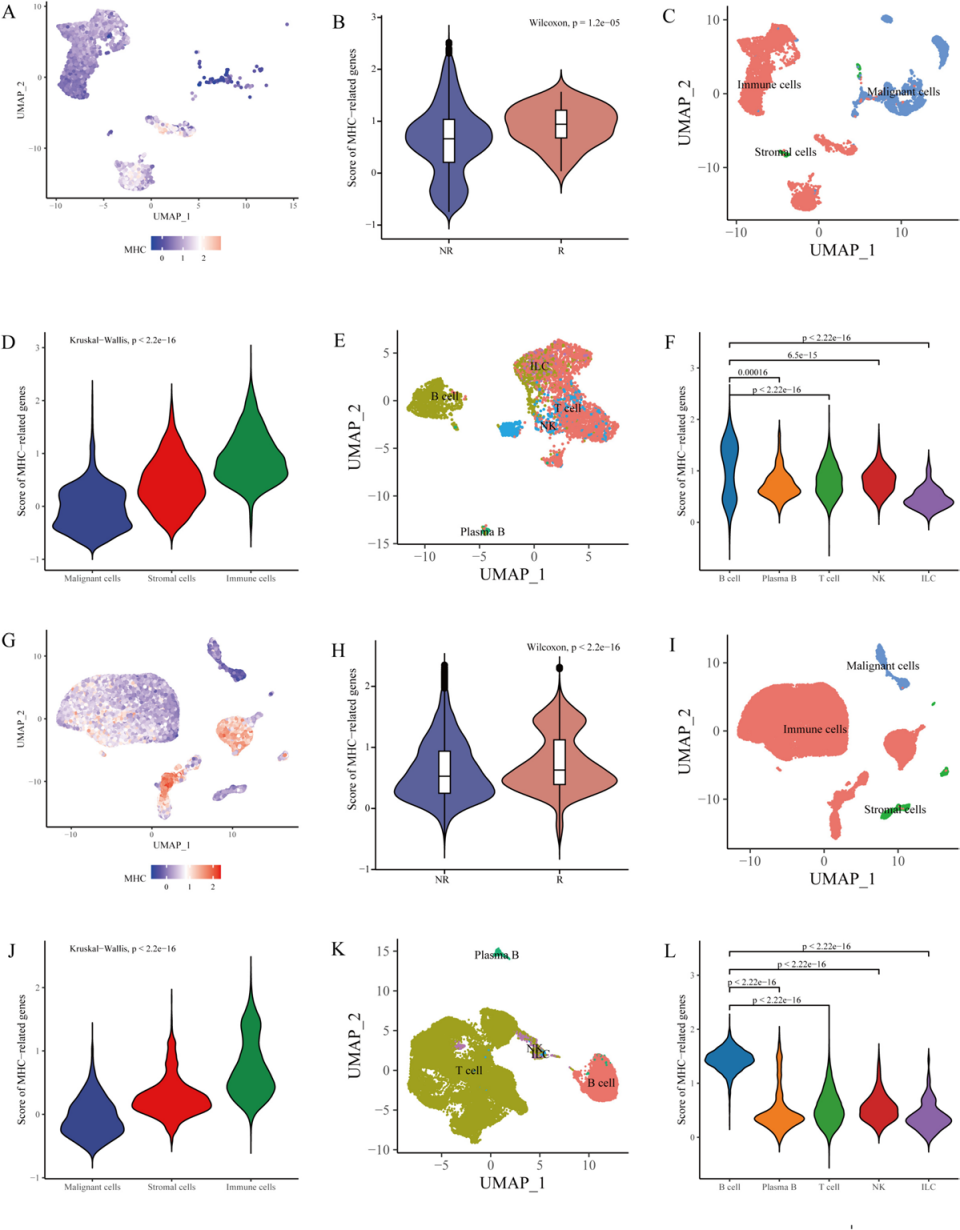
MHC-related gene score and immunotherapy in GSE115978 and GSE123813. **A** UMAP projection of MHC-related gene score of each cell in GSE115978. **B** MHC-related gene scores in non-responders and responders in GSE115978. **C** UMAP of cell subtypes in GSE115978. **D** MHC-related gene scores in different cell subtypes in GSE115978. **E** UMAP of cell subtypes in immune cells in GSE115978. **F** MHC-related gene scores in different immune cell subtypes in GSE115978. **G** UMAP projection of MHC-related gene score of each cell in GSE123813. **H** MHC-related gene scores in non-responders and responders in GSE123813. **I** UMAP of cell subtypes in GSE123813. **J** MHC-related gene scores in different cell subtypes in GSE123813. **K** UMAP of cell subtypes in immune cells in GSE123813. **L** MHC-related gene scores in different immune cell subtypes in GSE123813

In this study, we revealed and verified the negative association between MHC and immunotherapy outcomes in two single-cell RNA sequencing immunotherapy cohorts. Thereafter MHC-related gene signature (MHCsig) was developed through an integrative analysis of 34 single-cell RNA sequencing datasets. The predictive value of MHCsig was further explored and validated through a comprehensive analysis of pan-cancer transcriptomic data and independent immunotherapy cohorts. Our findings uncovered the potential of MHCsig for predicting immunotherapy outcomes, especially in low-grade glioma (LGG) (Fig. 1).

## Materials and methods

### Data source and processing

GSE115978 (melanoma) and GSE123813 (basal cell carcinoma) were downloaded from the Gene Expression Omnibus database. 34 single-cell RNA sequencing datasets were collected from TISCH, including malignant and stromal/immune cell data. The *Seurat* (version 4.0.6) package was used for single-cell RNA sequencing data processing as described previously [12].

Transcriptome data from The Cancer Genome Atlas (TCGA) pan-cancer cohort was downloaded from the UCSC Xena data portal to explore the potential of MHCsig and immunosuppression in 30 different cancer types. The total mutation burden (TMB) came from cBioPortal, and the intratumor heterogeneity (ITH) data came from Torsson et al. [13], these data were used to analyze the correlation with MHCsig.

A total of 10 immune checkpoint inhibitor bulk RNA sequencing cohorts with clinical information, including 5 SKCM cohorts, 2 urothelial carcinoma cohorts, 1 pleomorphic cohort Glioblastoma cohort, 1 gastric cancer cohort and 1 renal cell carcinoma cohort, were systematically collected.

A total of seven published CRISPR/Cas9 screening studies that assessed the individual impact of each gene knockout on tumor immunity were collected and grouped into 17 datasets according to the model cells and applied therapeutic conditions.

Somatic mutation data of LGG from TCGA were downloaded from the Genome Data Commons data portal.

### MHC-related genes acquisition

MHC-related genes were obtained from GeneCards and Molecular Signatures Database with the keyword of “MHC”. The obtained results were intersected to obtain the final MHC-related genes.

### Relationship between MHC-related genes and immunotherapy

Two datasets with clear tumor immunotherapy efficacy GSE115978 (melanoma) and GSE123813 (basal cell carcinoma) were used to explore the relationship between MHC-related genes and tumor immunotherapy. Patients were divided into two groups according to their response status evaluated by Response Evaluation Criteria in Solid Tumors (version 1.1): complete response and partial response as responders, or stable disease and progressive disease as non-responders.

### MHCsig acquisition

In this study, 34 single-cell sequencing datasets were included to screen MHCsig. We analyzed the relationship between MHC-related genes and their MHC scores in malignant tumor cells by Spearman correlation. Then, gene ontology enrichment analysis was used to show the biological function pathways of highly enriched MHCsig.

### Relationship between MHCsig and immune response

In this study, the correlation analysis between MHCsig and 75 immune-related genes [13] (Table S1) was carried out. We then assessed the infiltrating abundance of immune cells in pan-cancer to better characterize the immune microenvironment in different tumors. B cells could favorably affect immune checkpoint inhibitor response via tertiary lymphoid structure [14], next, we analyzed the relationship between tertiary lymphoid structure-related genes and MHCsig in the TCGA dataset pan-cancer dataset.

In addition, we analyzed the relationship between MHCsig and immune-relevant factors (ITH and TMB) with the *MCPcounter* package. Then, we compared the abundance of immune cells among these patients.

### MHCsig model construction

We collected 10 immune checkpoint inhibitor bulk RNA sequencing data cohorts with clinical information to investigate the predictive value of MHCsig. Patients received anti-PD-1/PD-L1, anti-CTLA-4, or anti-PD-1/PD-L1 plus anti-CTLA-4. All these 10 cohorts were split into 2 cohorts: the training cohort (80%, *N* = 618) and the validation cohort (20%, *N* = 154).

We used eight common machine learning algorithms. After training, eight models were harvested in the validation cohort and the model with the highest area under the receiver operating characteristic curve was selected as the MHCsig model.

### Screening of potential therapeutic targets for MHCsig

We collected data from seven published CRISPR/Cas9 screening studies to explore potential therapeutic targets for the MHCsig, as described previously [15].

### Hub-MHCsig acquisition

We used different machine learning algorithms to further screen the Hub-MHCsig, which was related to immune efficacy in the training cohort.

### The landscape of Hub-MHCsig among cancers

The scores of Hub-MHCsig in 30 cancers in the TCGA dataset were evaluated by ssGSEA analysis. Then, we reported the correlation between the scores of Hub-MHCsig and the abundance of immune cell infiltration and microsatellite instability among cancers.

#### Survival analysis

The *survival* package was used for survival analysis, the Kaplan–Meier survival curve was used to show the difference in survival, and the log-rank test was used to evaluate the significance of the difference in survival time between two groups of patients, which were defined by the *survminer* package.

#### LGG-related Hub-MHCsig acquisition

We ranked the 19 Hub-MHCsig by the random forest model and obtained the importance of each Hub-MHCsig. Hub-MHCsig with importance greater than 0 were considered as LGG-related Hub-MHCsig.

#### Construction of the prognostic model in LGG

In order to explore the relationship between LGG-related Hub-MHCsig and the prognosis of patients with LGG, we constructed the prognostic model through 5 machine learning algorithms. The *ConsensusClusterPlus* package was used to identify distinct functional phenotype characteristic populations. Finally, we compared the overall survival between clusters.

Based on the above analysis results, the nomogram was drawn by the *rms* package. Next, the calibration curve was used to evaluate the accuracy and resolution of the nomogram.

#### Somatic mutation heterogeneity in LGG

Somatic mutation data of LGG with all non-synonymous mutations were used for downstream analysis. The mutation status of LGG-related Hub-MHCsig was displayed by the *maftools* package.

#### Druggability estimation

Druggability data of anti-tumor compounds across tumor cell lines were collected from the Drug Gene Interaction database and were estimated by the *oncoPredict* package. Then, LGG-related Hub-MHCsig with logFC < 6 were selected to build the LGG-related Hub-MHCsig-drug network.

#### Immune infiltration quantification

The infiltration levels of immune cell compositions were scored and compared between groups by employing CIBERSORT and correlations between immune cell compositions were then estimated.

#### LGG-related Hub-MHCsig-miRNA/transcription factor network construction

The miRNA and transcription factor of LGG-related Hub-MHCsig were obtained from TargetScan and ENCODE, respectively. All data were uploaded to Cytoscape to build the networks.

## Experiment validation

### Cell culture

SVG p12, U-118MG, MCF-7, MDA-MB-231, SHSY-5Y, and SK-N-SH were obtained from National Collection of Authenticated Cell Cultures (Shanghai, China), U-138MG was obtained from the American Type Culture Collection (Manassas, VA, USA), and T98G was purchased from the FuHeng Cell Center (Shanghai, China). SHSY-5Y, and SK-N-SH were cultured in 1:1 mixture of EMEM (ATCC, Manassas, VA, USA) and Ham’s F12 Medium (Gbico, Grand Island, NY, USA) supplemented with 10% fetal bovine serum (Ozfan, Nanjing, China) and other cells were maintained in DMEM supplemented with 10% fetal bovine serum at 37 °C in a humidified atmosphere of 5% CO_2_.

#### RNA extraction and real-time quantitative PCR (RT-qPCR)

Total RNA was isolated by using the TRizol reagent (Invitrogen, USA) according to the manufacturer’s protocol. The extracted RNA was transcribed into cDNA using HiScript II Q Select RT SuperMix for qPCR(+ gDNA wiper) (Vazyme, Nanjing, China) and then quantitated by Taq Pro Universal SYBR qPCR Master Mix (Vazyme). The relative expression of indicated genes was normalized to *β*-actin with the comparative Ct method. The primer sequences are listed in Table S2.

#### Drug resistance experiments

Cells (5000 cells/well) were seeded in 96-well plates and after 24 h were incubated with Adriamycin (Selleck, Shanghai, China). The viability of the cells was determined using Celltiter-Glo Luminescent Cell Viability Assay (Promega, Beijing, China) according to the manufacturer’s instructions.

#### Clinical validation

We collected 125 LGG patients with survival data from Ren Ji Hospital, Shanghai Jiao Tong University School of Medicine (Shanghai, China). This study was approved by the Ethics Committee of Ren Ji Hospital, Shanghai Jiao Tong University School of Medicine (RA-2022–052). The expression of 8 LGG-related Hub-MHCsig (B2M, HLA-B, HLA-DOA, HLA-DPB1, HLA-DRB1, HLA-E, TAP1, and TAP2) was tested by immunohistochemistry, as described previously [16]. The details of antibody are shown in Table S3. All patients were divided into high expression and low expression groups based on the median expression of this LGG-related Hub-MHCsig in each sample. Survival analysis was performed using *survival* package (version 3.5–7), and the significance of survival differences between the two groups was calculated using the log rank test, survival curves for each gene were plotted using the *survminer* package (version 0.4.9).

#### Statistical analysis

All data processing and analysis were done by *R* (version 4.0.2) and GraphPad Prism (version 9.5). For the comparison of two groups of continuous variables, the statistical significance of normally distributed variables was estimated by an independent Student *t*-test, and the difference between non-normally distributed variables was analyzed by the Mann–Whitney *U* test. The Chi-square test or Fisher’s exact test was used to compare and analyze statistical significance between two groups of categorical variables. *P* < 0.05 was used as the criterion for significant difference results.

## Results

### MHC-related genes acquisition

We retained a total of 65 and 21 MHC-related genes from GeneCards and Molecilar Signatures Database, respectively. After the intersection, 21 MHC-related genes were finally obtained (Table S4).

### MHC-related genes were associated with immunotherapy

A total of 32 patients were included in the GSE115978 after quality control, including 15 patients in the immunotherapy ineffective group, 16 patients in the non-immunotherapy group, and 1 patient in the immunotherapy effective group. The MHC-related gene score of each cell is shown in Fig. 1A. It was found that the MHC-related gene scores in the immunotherapy-noneffective group were lower than those in the immunotherapy-effective group (*P* < 0.0001) (Fig. 1B). From these cells, we mainly confirmed three cell subtypes, namely immune cells, malignant cells, and stromal cells (Fig. 1C). Among them, immune cells had the highest MHC-related gene scores (*P* < 0.0001) (Fig. 1D). Furthermore, immune cells were identified with B cells, T cells, plasma B, innate lymphoid cells, and natural killer cells (Fig. 1E), and B cells had the highest MHC-related gene scores (Fig. 1F).

Similarly, GSE123813 included 10 patients after quality control, including 6 patients with effective immunotherapy and 4 patients with ineffective immunotherapy. The MHC-related gene score of each cell is shown in Fig. 1G. It was found that the MHC-related gene scores in the immunotherapy-noneffective group were lower than those in the immunotherapy-effective group (*P* < 0.0001) (Fig. 1H). From these cells, we mainly confirmed three cell subtypes, namely immune cells, malignant cells, and stromal cells (Fig. 1I). Among them, immune cells had the highest MHC-related gene scores (*P* < 0.0001) (Fig. 1J). Furthermore, immune cells were identified with B cells, T cells, plasma B, innate lymphoid cells, and natural killer cells (Fig. 1K), and B cells had the highest MHC-related gene scores (Fig. 1L).

### MHCsig acquisition

**Fig. 2:**
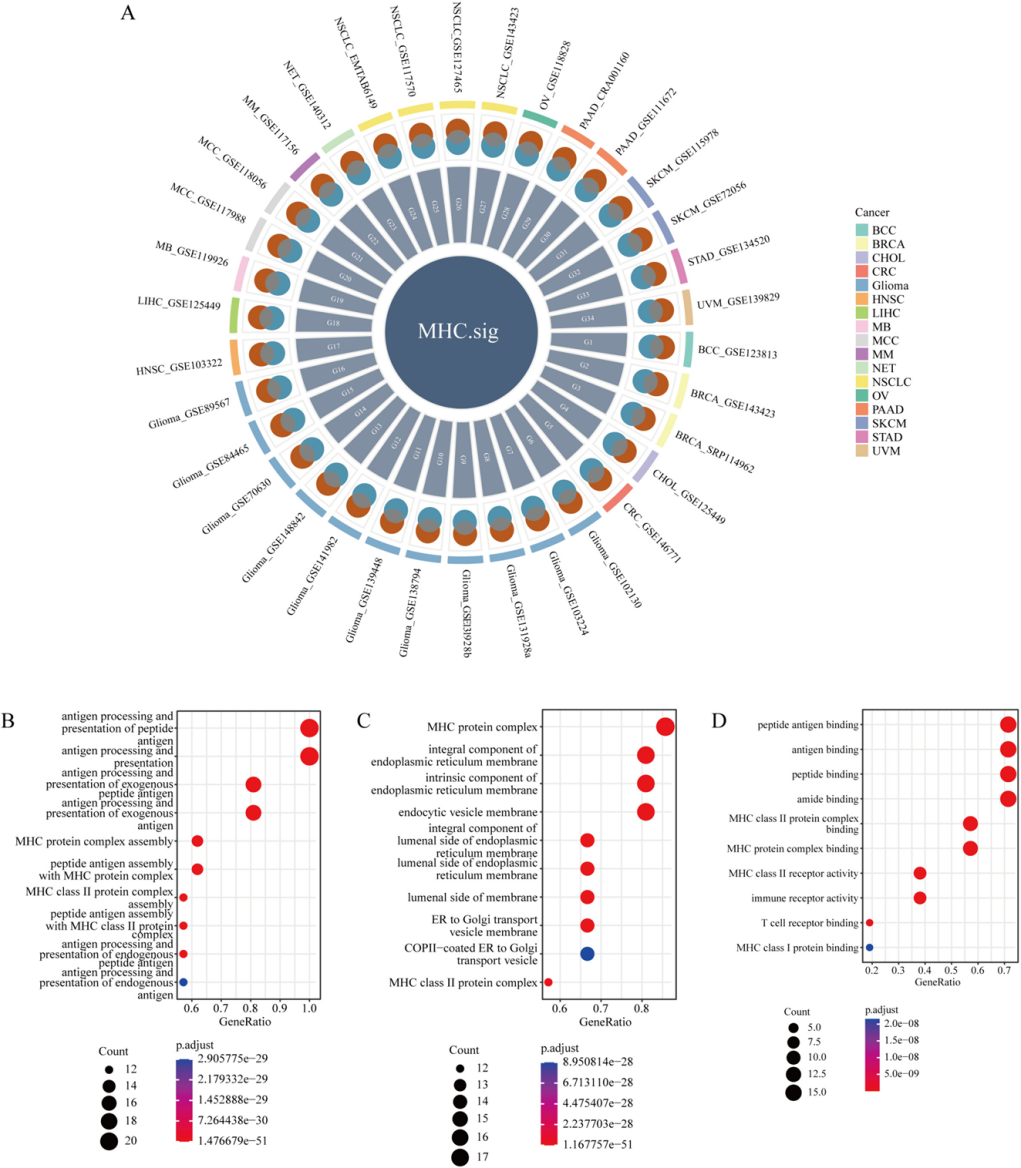
The enrichment of MHCsig among different cell subtypes. **A** Relationship between MHCsig and their MHC scores in malignant tumor cells. **B** Biological function enrichment analysis of MHCsig. **C** Cellular function enrichment analysis of MHCsig. **D** Molecular function enrichment analysis results of MHCsig

Since MHC-related genes were associated with drug resistance to tumor immunotherapy [17, 18], we hypothesized that the level of MHC-related genes in patients could predict the efficacy of immunotherapy. We obtained MHCsig from 34 single-cell RNA sequencing datasets (Fig. 2A). Functional analysis showed that MHCsig were mainly enriched in antigen processing and presentation of peptide antigen, antigen processing and presentation and antigen processing and presentation of exogenous peptide antigen and other biological processes (Fig. 2B), MHC protein complex, integral component of endoplasmic reticulum membrane and endocytic vesicle membrane and other cellular components (Fig. 2C), as well as peptide antigen binding, antigen binding, and peptide binding (Fig. 2D).

### MHCsig was positively correlated with immune response and infiltration

**Fig. 3:**
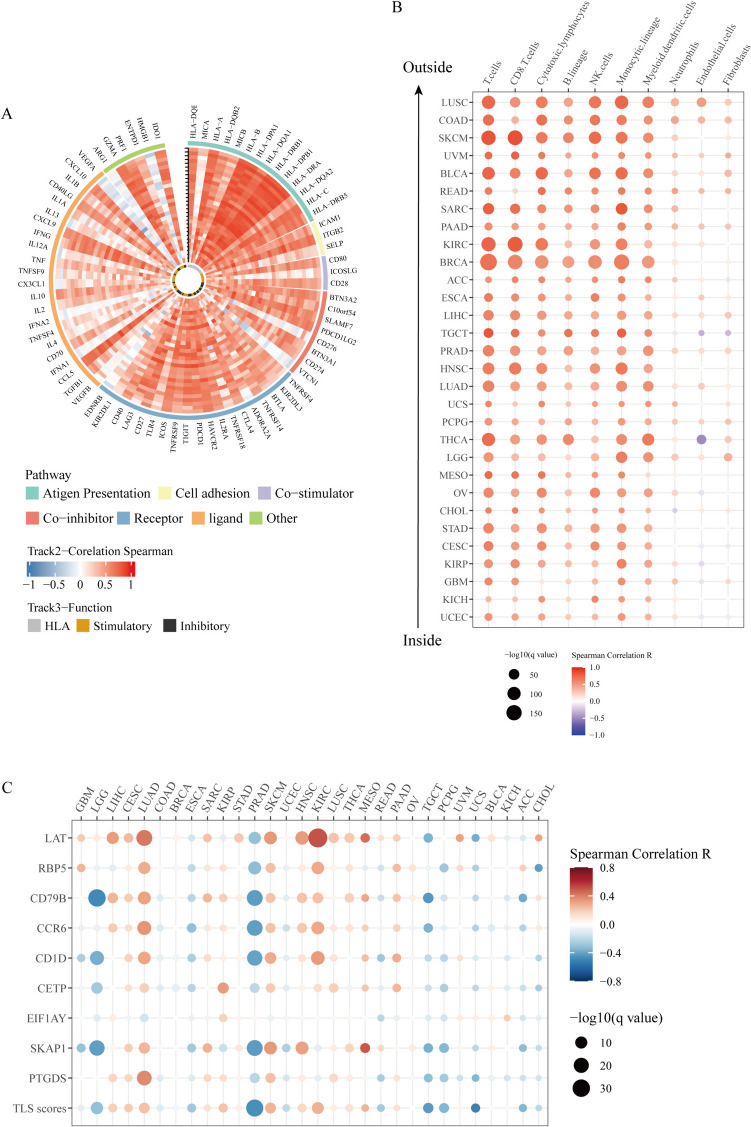
MHCsig was positively correlated with immune response. **A** Expression levels of MHCsig and immune checkpoint inhibition-related genes. **B** Correlation of expression levels of MHCsig and immune checkpoint inhibition-related genes. **C** Correlation between MHCsig and tertiary lymphoid structure-related genes

MHCsig were positively correlated with most immune-related genes (Fig. 3A). Moreover, MHCsig enhanced the infiltration of various immune-promoting cells in the immune microenvironment of different cancer types (Fig. 3B), indicating MHCsig was positively correlated with tumor immune response. We found that some tertiary lymphoid structure-related genes such as LAT, RBP5, SKAP1, and CCR6 were positively correlated with MHCsig in pan-cancer (Fig. 3C).

**Fig. 4:**
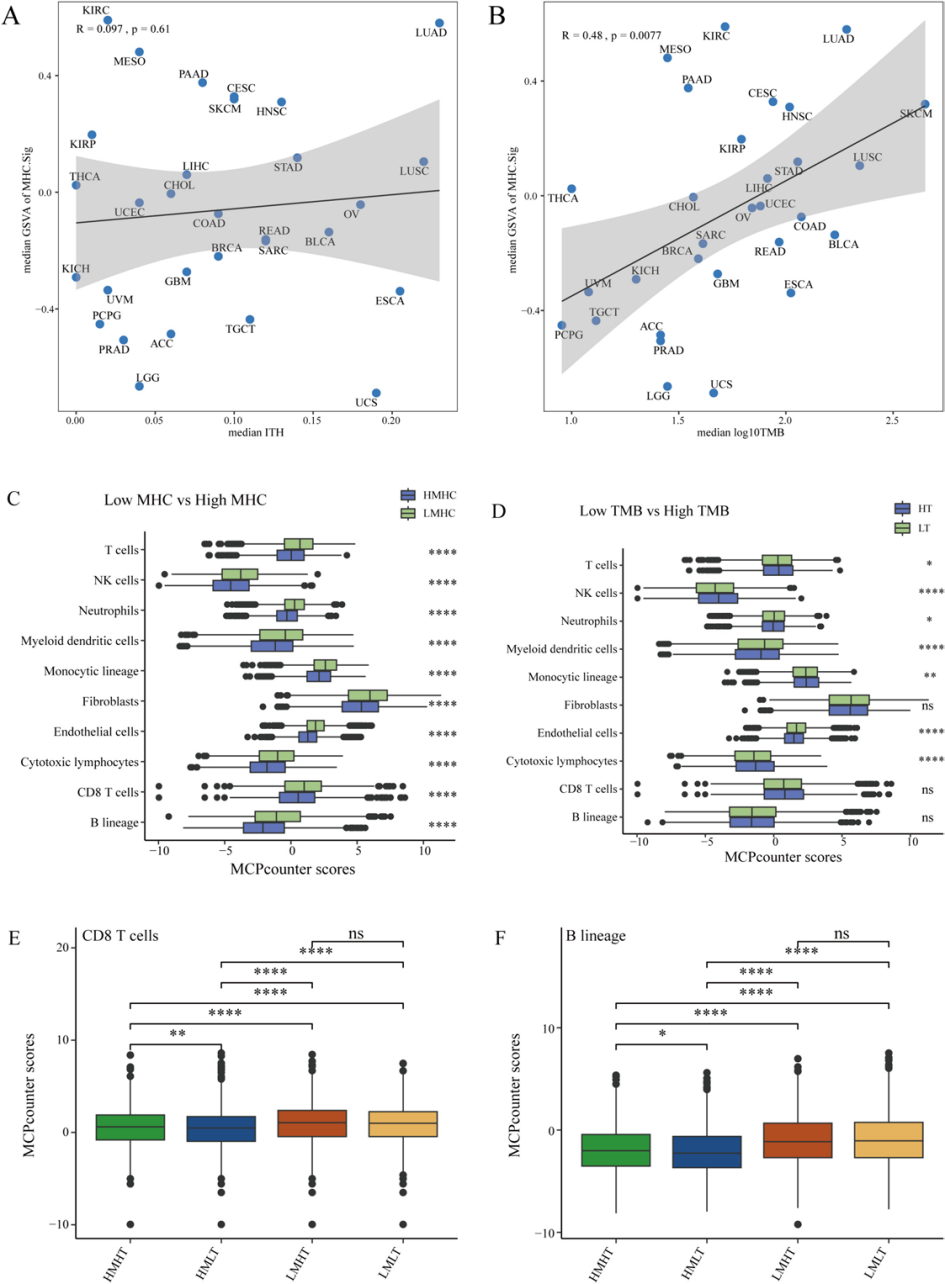
MHCsig was positively correlated with immune infiltration. **A** Correlation of median MHCsig and median TMB of each cancer type. **B** Correlation of median MHCsig and median ITH of each cancer type. **C** Immune infiltrating in MHCsig. **D** Immune infiltrating in TMB. **E** CD8 T cells infiltrating in MHCsig and TMB. **F** B lineage infiltrating in MHCsig and TMB. HMHT, high MHCsig/high TMB; HMLT, high MHCsig/low TMB; LMHT, low MHCsig/high TMB; LMLT, low MHCsig/low TMB

In addition, we analyzed the relationship between MHCsig and immune-relevant factors (ITH and TMB). It was found that there was no statistically significant correlation between ITH and MHCsig in pan-cancer (*R* = 0.097, *P* = 0.61, Fig. 4A). Interestingly, TMB was positively correlated with MHCsig in pan-cancer (*R* = 0.48, *P* < 0.01, Fig. 4B). So we further estimated the population abundance of tissue-infiltrating immune and stromal cell populations regardless of MHCsig and TMB. It was found that all immune and stromal cells were differently infiltrated in MHCsig (Fig. 4C). T cells, natural killer cells, neutrophils, myeloid dendritic cells, monocytic lineage, endothelial cells, and cytotoxic lymphocytes were differently infiltrated in TMB while CD8 T cells, B lineage, and fibroblasts were not differently infiltrated in TMB (Fig. 4D). Furthermore, CD8 T cells and B lineage were differently infiltrated in high MHCsig regardless of TMB (Fig. 4E & F), indicating high MHCsig was more immune sensitive than low MHCsig regardless of TMB level.

### MHCsig predicted immunotherapy outcome

We got 8 models after training in the training cohort, and the logistic regression model had the highest AUC in the validation cohort, with a value of 0.68 (Fig. S1A & B). Therefore, the logistic regression model was selected as the MHCsig model.

### Potential therapeutic targets screened from MHCsig

A total of 22,505 genes were obtained from 17 datasets, of which 563 and 1350 were the 2.5% and 6% top-ranked genes number, respectively. A total of 8 top-ranked genes and 8 bottom-ranked genes representing immune-resistant genes and immune-sensitive genes are shown in Fig. S2A. Then we found that MHCsig had the lowest percentage of top-ranked genes than other immune signatures (Fig. S2B). The top 6% immune-resistant genes included 5 MHCsig (HLA-DOA, HLA-DMB, HLA-DQA1, HLA-DMA, and HLA-DRB) and were down-represented in multiple independent CRISPR/Cas9 datasets (Fig.S2C). Importantly, 5 MHCsig were upregulated in the breast cancer cells and neuroblastoma cells with high malignancy (Fig. S2D & E), which might serve as potential therapeutic targets in synergy with immune checkpoint inhibitors.

### Hub-MHCsig acquisition

In this study, five different algorithms were applied and intersected. The results show that there were 19 Hub-MHCsig, namely, B2M, HLA-A, HLA-B, HLA-C, HLA-DMA, HLA-DMB, HLA-DOA, HLA-DOB, HLA-DPA1, HLA-DPB1, HLA-DQA1, HLA-DQB1, HLA-DRA, HLA-DRBI, HLA-E, HLA-F, TAP1, TAP2, and TAPBP.

### The landscape of Hub-MHCsig among different tumor types

In this study, the scores of Hub-MHCsig in 30 cancers in the TCGA dataset were first evaluated by ssGSEA analysis (Fig. S3A). Then we analyzed the correlation between Hub-MHCsig and the abundance of immune cell infiltration in different cancer types and found that M1 macrophages, CD8 T cells, and regulatory T cells were positively correlated with the scores of Hub-MHCsig in all cancers while M0 macrophages, resting NK cells, and activated dendritic cells were negatively correlated with the scores of Hub-MHCsig in most cancers (Fig. S3B). In some cancers, the scores of Hub-MHCsig were positively correlated with microsatellite instability while the scores of Hub-MHCsig were negatively correlated with microsatellite instability in cholangiocarcinoma, lung adenocarcinoma, and pancreatic cancer (Fig. S3C). Moreover, among 19 Hub-MHCsig, HLA-DMA, HLA-DMB, and HLA-DOA were positively correlated with microsatellite instability in colon adenocarcinoma, while TAP2, TAP1, and HLA-F were negatively correlated with microsatellite instability in testicular germ cell tumors (Fig. S3D).

### Hub-MHCsig was associated with multiple tumor prognoses

We subsequently performed survival analysis across tumor types. Hub-MHCsig was associated with pan-cancer overall survival (Fig. S4A) and disease-special survival (Fig. S4A). Higher Hub-MHCsig was associated with worse overall survival (*P* < 0.0001, Fig. S4B) and disease-special survival (*P* < 0.001, Fig. S4C) in LGG.

#### LGG-related Hub-MHCsig acquisition

In this study, the 19 Hub-MHCsig were ranked by the random forest model. It can be seen that the error rate of describing the survival of LGG was decreasing with the inclusion of different Hub-MHCsig (Fig. S5A). In order to screen the LGG-related Hub-MHCsig, Hub-MHCsig with importance greater than 0 were selected. Finally, 10 LGG-related Hub-MHCsig (B2M, HLA-B, HLA-C, HLA-DOA, HLA-DPB1, HLA-DRA, HLA-E, TAP1, TAP2, and TAPBP) were obtained (Fig. S5B).

#### LGG-related Hub-MHCsig was related to survival

**Fig. 5:**
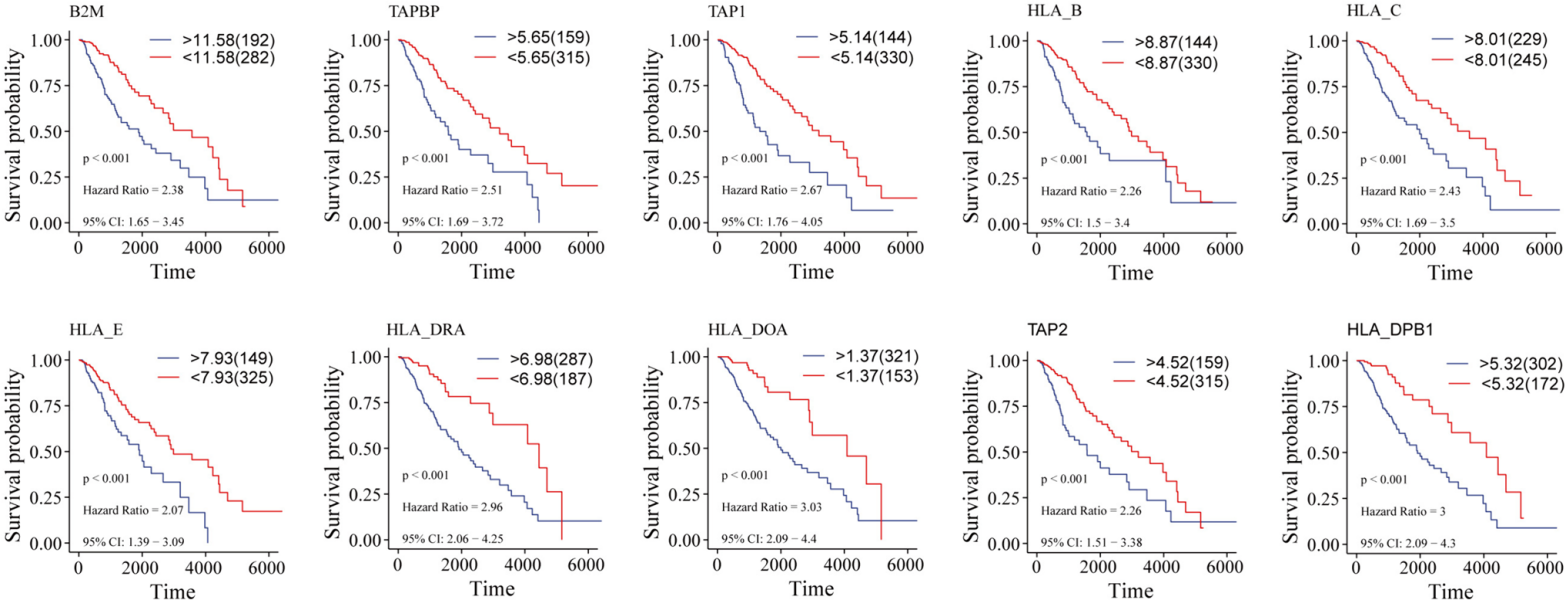
Overall survival analysis of 10 LGG-related Hub-MHCsig in LGG

Next, we evaluated the relationship between 10 LGG-related Hub-MHCsig and survival in patients with LGG. The patients with high expression of each 10 LGG-related Hub-MHCsig had worse overall survival (all *P* < 0.001, Fig. 5) and disease-special survival (all *P* < 0.001, Fig. S6) than those with low expression of that.

**Fig. 6:**
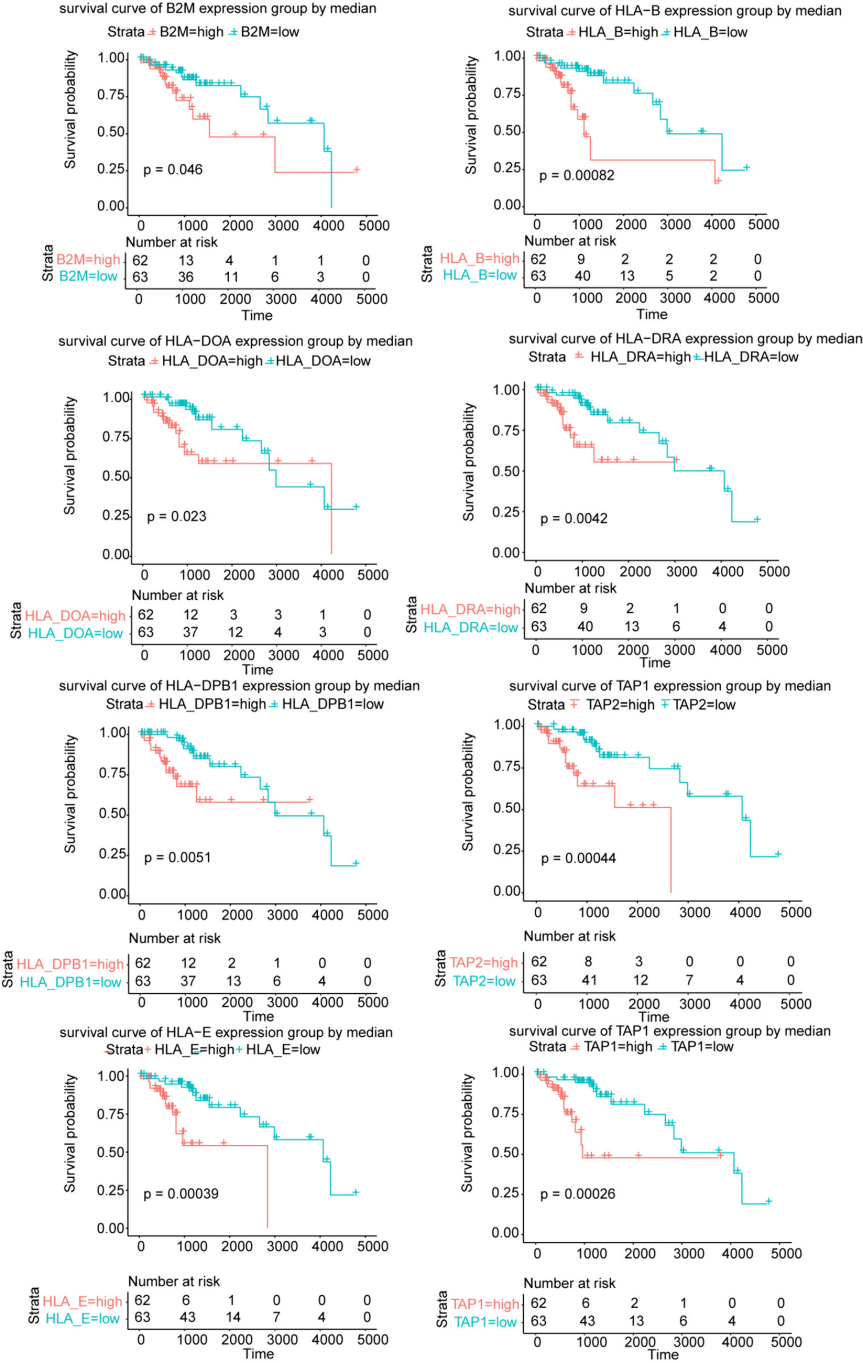
Overall survival analysis of 8 LGG-related Hub-MHCsig in LGG from our clinical data

The expression of 10 LGG-related Hub-MHCsig was then validated in LGG cell lines and we found that 8 LGG-related Hub-MHCsig (B2M, HLA-B, HLA-DOA, HLA-DPB1, HLA-DRB1, HLA-E, TAP1, and TAP2) were upregulated in LGG (Fig. S7). Furthermore, the expression of 8 LGG-related Hub-MHCsig was validated in our clinical LGG patients (Fig. S8), and we found that patients with high expression of each 8 LGG-related Hub-MHCsig had worse overall survival (all *P* < 0.05, Fig. 6).

#### Construction of the prognostic model in LGG

In the TCGA-BLCA dataset, we used 10 LGG-related Hub-MHCsig to construct a prognostic model. We found that the prediction performance of the prognostic models was all high (C-index > 0.75), and the prognostic model constructed by the StepCox was the best (C-index = 0.8386, Fig. S9A). Cumulative distribution function indicated TCGA-BLCA could be divided into two clusters (Fig. S9B), then, two consensus clusters (ClusterA and ClusterB) were identified (Fig. S9C). Principal co-ordinates analysis demonstrated the classification stability was high (Fig. S9D). Finally, patients in ClusterA had better overall survival than those in ClusterB (*P* < 0.001, Fig. S9E).

We built the nomogram to predict the 1-year, 3-year, and 5-year survival probability in patients with LGG (Fig. S10A). The calibration curves indicated the nomogram goodness-of-fit test (Fig. S10B ~ D). The time-dependent ROC curves indicated the AUCs of the prognostic model for the 1-year, 3-year, and 5-year prognosis of patients with LGG were 0.78 (70.51–85.45), 0.76 (68.68–83.02), and 0.69 (59.8–77.39), respectively (Fig. S10E).

#### Somatic mutation characteristics and druggability of LGG-related Hub-MHCsig

**Fig. 7:**
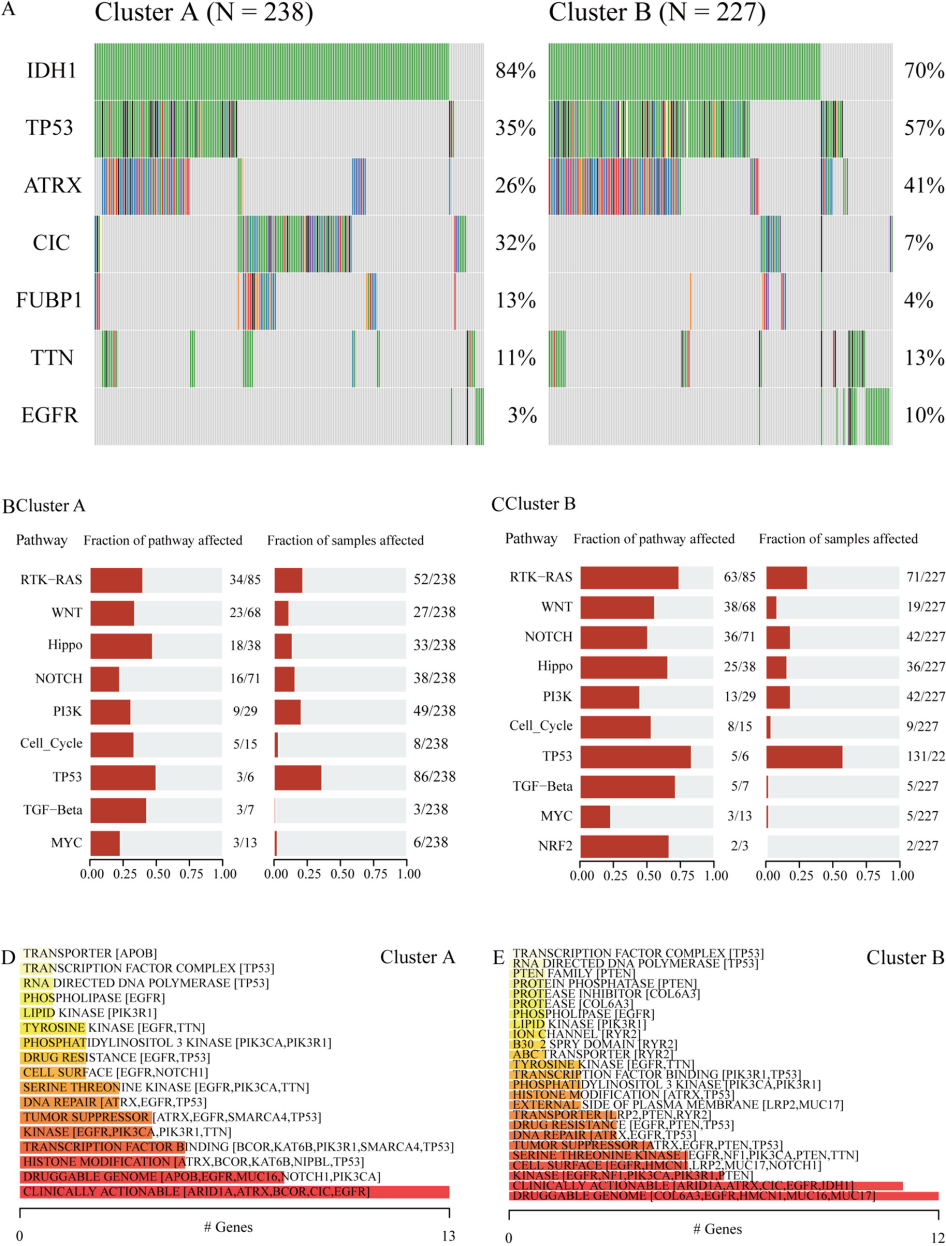
Somatic mutation characteristics and druggability of LGG-related Hub-MHCsig. **A** LGG-related Hub-MHCsig mutation landscape in ClusterA and ClusterB. **B** Biological functions affected by mutations in ClusterA. **C** Biological functions affected by mutations in ClusterB. **D** Classification of potentially druggable genes in ClusterA. **E** Classification of potentially druggable genes in ClusterB. The top 5 genes in square brackets after each classification are displayed. If there are less than 5 genes, all are displayed. The Y-axis is the number of genes in the druggable gene category

Somatic mutation frequencies were investigated. Overall, IDH1, TP53, ATRX, CIC, FUBP1, TTN, and EGFR mutation frequencies were high in LGG, among which the mutated frequency of IDH1 was 84% in ClusterA and 70% in ClusterB (Fig. 7A). The biological function changes caused by mutations in ClusterA were mainly concentrated in the TP53 and Hippo signaling pathways (Fig. 7B), and the biological function changes caused by the mutations in ClusterB were mainly concentrated in the TP53 and RTK-RAS signaling pathways (Fig. 7C). Next, the mutations of the two clusters of LGG were analyzed to explore the druggability of the gene and the interaction between the drug and the gene, and we found that genes that predict the drug might act on ClusterA and ClusterB were clinically actionable (Fig. 7D) and druggable genome (Fig. 7E), respectively. Through logFC < 6 filtering, 5 (PIK3R3, KCNA6, TARDBP, RGS5, and TOP2A) and 4 (PIK3R3, KCNA6, TARDBP, and RGS5) LGG-related Hub-MHCsig were left in ClusterA and ClusterB, respectively, and the LGG-related Hub-MHCsig-drug networks are shown in Fig. S11. Importantly, these 5 LGG-related Hub-MHCsig in ClusterA and ClusterB were upregulated in the LGG cells with high malignancy (Fig. S12A), and the half maximal inhibitory concentration of Adriamycin in U-118MG and T98G was 9.93 (8.14 ~ 12.36) μM and 55.85 (50.09 ~ 62.97) μM, respectively (Fig. S12B).

#### Immune infiltration characteristics in LGG

**Fig. 8:**
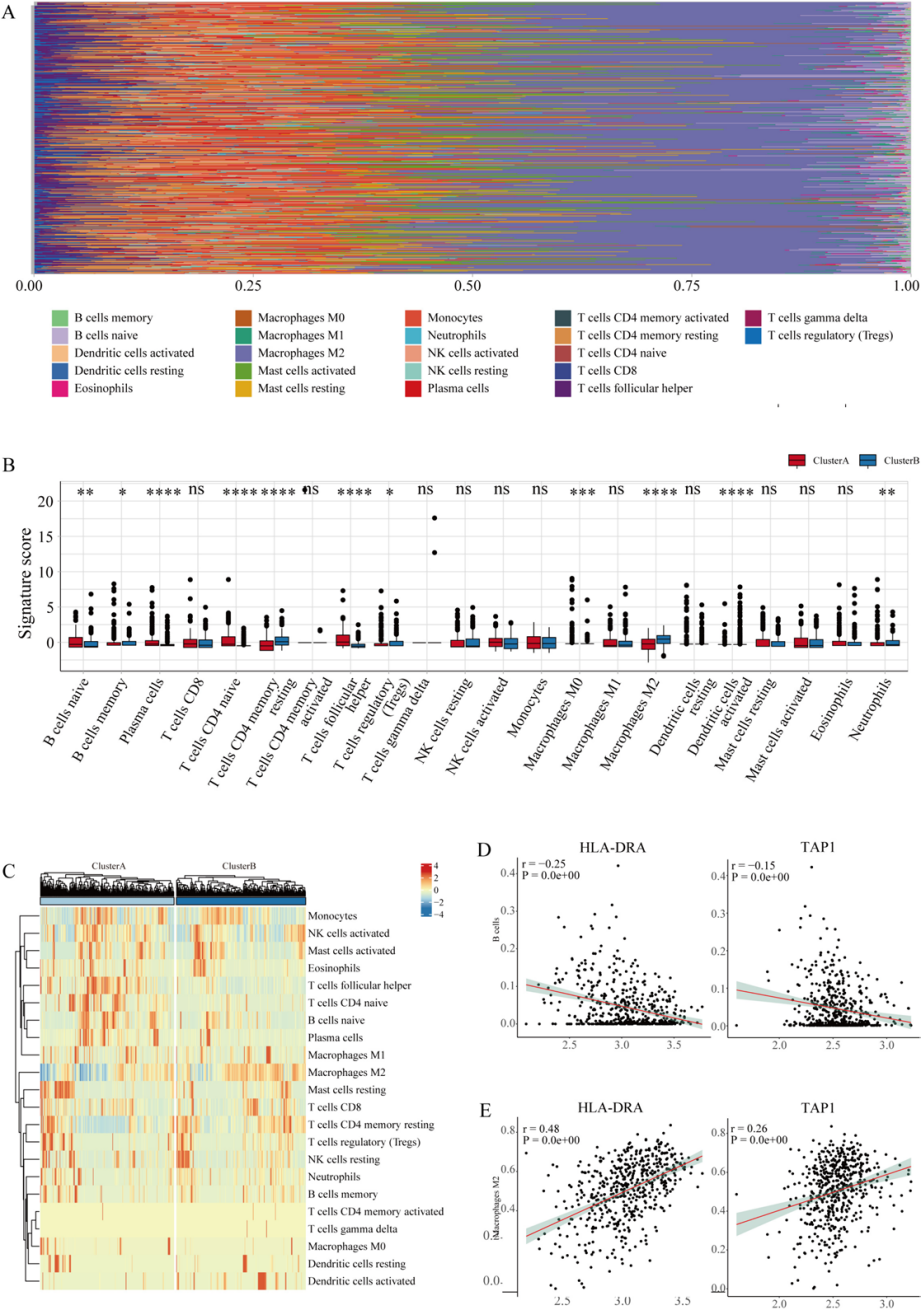
Immune cell infiltration analysis. **A** Histogram of the proportion of immune cells in the TCGA-LGG dataset. **B** Box plot of immune cell infiltration abundance in LGG. **C** Heat map of immune cell infiltration abundance in TCGA-LGG dataset. **D** HLA-DRA and TAP1 were negatively correlated with B cells. **E** HLA-DRA and TAP1 were positively correlated with M2 macrophage

We scored the infiltration of 22 immune cell compositions across LGG (Fig. 8A). The infiltration abundance of activated B cells, activated CD4 cells, and plasma cells in ClusterA was significantly higher than that in ClusterB (Fig. 8B). The infiltration abundance of memory B cells, resting memory CD4 T cells, and regulatory T cells in ClusterB was significantly higher than that in ClusterA (Fig. 8B). LGG-related Hub-MHCsig was differently expressed in immune cells (Fig. 8C). HLA-DRA and TAP1 were negatively correlated with B cells (*R* = −0.25, *P* < 0.001, and *R* = −0.15, *P* < 0.001, respectively) (Fig. 8D) and HLA-DRA and TAP1 were positively correlated with M2 macrophage (*R* = 0.48, *P* < 0.001, and *R* = 0.26, *P* < 0.001, respectively) (Fig. 8E).

#### Regulatory network construction

We obtained 58 miRNAs for 10 LGG-related Hub-MHCsig (Fig. S13A). We obtained 71 transcription factors for 10 LGG-related Hub-MHCsig (Fig. S13B).

## Discussion

The mechanism between MHC and immunotherapy has been widely explored and targeting MHC could serve as an opportunity for cancer immunotherapy [19], however, direct clinical evidence on the association of MHC and immunotherapy response has not been reported. Here, we obtained 21 MHC-related genes and then evaluated the MHC-related genes of individual malignant cells and uncovered the inverse correlation between MHC-related genes and immunotherapy outcomes. Antigenic epitopes presented by MHC molecules to induce tumor-specific T cells. Based on this, MHC might have a certain role in pan-cancer. So we carefully validated the predictive value of MHCsig in various cancers. Remarkably, MHCsig achieved better performance in LGG. We later distinguished the performance of MHCsig and potential molecular mechanisms. This study is the first report to demonstrate the robust link between MHCsig and immunotherapy outcomes. Most importantly, we constructed Hub-MHCsig, which successfully predicts response to immunotherapy across multiple cancer types, and primarily explored the regulatory networks in LGG.

We found that MHCsig were mainly enriched in antigen processing and presentation. Therefore, MHC could respond to immunotherapy [20]. Furthermore, we evaluated the correlation between MHCsig and immune infiltration. Additionally, we found MHCsig had a positive statistically significant correlation with TMB in pan-cancer. High MHCsig is associated with high TMB, which is a predictor for benefit from immune checkpoint blockade treatment [21]. Although TMB is a recognized immunotherapy biomarker, there are still a considerable number of patients with high TMB who do not respond to immunotherapy [22]. Our stratified analysis revealed that high MHCsig was more immune sensitive than low MHCsig regardless of TMB level. Considering such a robust link between MHCsig and immunotherapy outcomes, we explored potential drug targets from MHCsig. MHCsig had a low percentage of top-ranked genes, namely immune-resistant genes. Five MHCsig (HLA-DOA, HLA-DMB, HLA-DQA1, HLA-DMA, and HLA-DRB) showed immune-resistant characteristics. HLA-DM is a potential novel target for cellular and immunotherapy of leukemia [23], which is consistent with the results we obtained. These MHCsig might serve as potential therapeutic targets among various cancers and further research on these MHCsig will help develop a combined immunotherapy strategy.

There are several limitations in our study. Firstly, it is not enough to evaluate immunotherapy response solely from two single-cell RNA sequencing datasets, although these are two independent datasets. Moreover, 10 immune checkpoint inhibitor bulk RNA sequencing cohorts did not cover all cancers, although we compensated with TCGA data containing 30 cancers. Thirdly, some datasets have incomplete clinical information, which to some extent weakens the reliability of our results. Moreover, although we validated the function and mechanism of MHCsig, more experiments about how MHCsig interacts with various components of the tumor microenvironment, such as immune cells, stromal cells, and other soluble factors, how MHCsig influences immune cell infiltration, activation, and function within the tumor microenvironment, as well as how MHCsig affects the expression of immune checkpoints or other immune-modulatory molecules are needed. Finally, although our analysis is relatively synthetical and comprehensive, it is only based on the publicly available data, and subsequent prospective clinical validation is necessary.

## Conclusions

To our knowledge, this is the first conclusive clinical evidence to be provided regarding the association between MHCsig and immunotherapy response. Our study demonstrates a promising solution for patient selection in immunotherapy and elucidates the use of targeted MHC to address to immunotherapy resistance.

### Supplementary Information

Below is the link to the electronic supplementary material.

Supplementary file1 (PDF 2861 kb)

## Data Availability

Publicly available datasets were analyzed in this study. This data can be found here: https://www.ncbi.nlm.nih.gov/geo; http://tisch.comp-genomics.org/; https://xenabrowser.net; https://www.cbioportal.org; https://portal.gdc.cancer.gov/; https://www.genecards.org; https://www.gsea-msigdb.org/gsea.

## References

[1] Forde PM, Spicer J, Lu S (2022). Neoadjuvant nivolumab plus chemotherapy in resectable lung cancer. N Engl J Med.

[2] Mirza MR, Chase DM, Slomovitz BM (2023). Dostarlimab for primary advanced or recurrent endometrial cancer. N Engl J Med.

[3] Wolchok JD, Chiarion-Sileni V, Gonzalez R (2022). Long-Term outcomes with nivolumab plus ipilimumab or nivolumab alone versus ipilimumab in patients with advanced melanoma. J Clin Oncol.

[4] Bagchi S, Yuan R, Engleman EG (2021). Immune checkpoint inhibitors for the treatment of cancer: clinical impact and mechanisms of response and resistance. Annu Rev Pathol.

[5] Horton R, Wilming L, Rand V (2004). Gene map of the extended human MHC. Nat Rev Genet.

[6] Zhang Z, Rohweder PJ, Ongpipattanakul C (2022). A covalent inhibitor of K-Ras(G12C) induces MHC class I presentation of haptenated peptide neoepitopes targetable by immunotherapy. Cancer Cell.

[7] Rodig SJ, Gusenleitner D, Jackson DG (2018). MHC proteins confer differential sensitivity to CTLA-4 and PD-1 blockade in untreated metastatic melanoma. Sci Transl Med.

[8] Yu J, Wu X, Song J (2022). Loss of MHC-I antigen presentation correlated with immune checkpoint blockade tolerance in MAPK inhibitor-resistant melanoma. Front Pharmacol.

[9] Roemer MG, Advani RH, Redd RA (2016). Classical Hodgkin lymphoma with reduced *β*2M/MHC class I expression Is associated with inferior outcome independent of 9p24.1 status. Cancer Immunol Res.

[10] Roemer MGM, Redd RA, Cader FZ (2018). Major histocompatibility complex class II and programmed death ligand 1 expression predict outcome after programmed death 1 blockade in classic Hodgkin lymphoma. J Clin Oncol.

[11] Tarhini AA, Lee SJ, Tan AC (2022). Improved prognosis and evidence of enhanced immunogenicity in tumor and circulation of high-risk melanoma patients with unknown primary. J Immunother Cancer.

[12] Liang Bo, Liang W-L, Liao H-L (2023). Single-cell and bulk characterisation of the distinct immune landscape and possible regulatory mechanisms in coronary plaques vulnerability. Clin Transl Med.

[13] Thorsson V, Gibbs DL, Brown SD (2018). The immune landscape of cancer. Immunity.

[14] Helmink BA, Reddy SM, Gao J (2020). B cells and tertiary lymphoid structures promote immunotherapy response. Nature.

[15] Zhang Z, Wang ZX, Chen YX (2022). Integrated analysis of single-cell and bulk RNA sequencing data reveals a pan-cancer stemness signature predicting immunotherapy response. Genome Med.

[16] Liang Bo, Zhang X-X, Li R (2022). Guanxin V protects against ventricular remodeling after acute myocardial infarction through the interaction of TGF-*β*1 and Vimentin. Phytomedicine.

[17] Ugurel S, Spassova I, Wohlfarth J (2019). MHC class-I downregulation in PD-1/PD-L1 inhibitor refractory Merkel cell carcinoma and its potential reversal by histone deacetylase inhibition: a case series. Cancer Immunol Immunother.

[18] James JL, Taylor BC, Axelrod ML (2023). Polycomb repressor complex 2 suppresses interferon-responsive MHC-II expression in melanoma cells and is associated with anti-PD-1 resistance. J Immunother Cancer.

[19] Karpiński P, Łaczmański Ł, Sąsiadek MM (2020). Major histocompatibility complex genes as therapeutic opportunity for immune cold molecular cancer subtypes. J Immunol Res.

[20] Zhou Y, Bastian IN, Long MD (2021). Activation of NF-κB and p300/CBP potentiates cancer chemoimmunotherapy through induction of MHC-I antigen presentation. Proc Natl Acad Sci USA.

[21] Negrao MV, Skoulidis F, Montesion M (2021). Oncogene-specific differences in tumor mutational burden, PD-L1 expression, and outcomes from immunotherapy in non-small cell lung cancer. J Immunother Cancer.

[22] Ready N, Hellmann MD, Awad MM (2019). First-line nivolumab plus ipilimumab in advanced non–small-cell lung cancer (CheckMate 568): outcomes by programmed death ligand 1 and tumor mutational burden as biomarkers. J Clin Oncol.

[23] Meurer T, Crivello P, Metzing M (2021). Permissive HLA-DPB1 mismatches in HCT depend on immunopeptidome divergence and editing by HLA-DM. Blood.

