# Supplementary material for: Integrative analysis of single-cell and bulk RNA sequencing unveils a machine learning-based pan-cancer major histocompatibility complex-related signature for predicting immunotherapy efficacy
Source: Cancer Immunol Immunother. 2024 May 7;73(7):121. doi: 10.1007/s00262-024-03714-5 (PMC11076435; doi:10.1007/s00262-024-03714-5)
Supplement: Supplementary file 1 — Supplementary file1 (PDF 2861 kb) [file 262_2024_3714_MOESM1_ESM.pdf]

Integrative Analysis of Single-Cell and Bulk RNA Sequencing Unveils a Machine Learning-Based Pan-Cancer Major Histocompatibility Complex-Related Signature for Predicting Immunotherapy Efficacy

Additional files

Table S1. List of 75 immune-related genes.

HLA-DQB1	MICA	HLA-A	HLA-DQB2	MICB	HLA-B	HLA-DPA1
HLA-DQA1	HLA-DRB1	HLA-DPB1	HLA-DRA	HLA-DQA2	HLA-C	HLA-DRB5
ICAM1	ITGB2	SELP	BTN3A2	C10orf54	SLAMF7	PDCD1LG2
CD276	BTN3A1	CD274	VTCN1	CD80	ICOSLG	CD28
VEGFB	TGFB1	CCL5	IFNA1	CD70	IL4	TNFSF4
IFNA2	IL2	IL10	CX3CL1	TNFSF9	TNF	IL12A
IFNG	CXCL9	IL13	IL1A	CD40LG	IL1B	CXCL10
VEGFA	ARG1	GZMA	PRF1	ENTPD1	HMGB1	IDO1
TNFRSF4	KIR2DL3	BTLA	TNFRSF14	ADORA2A	CTLA4	TNFRSF18
IL2RA	HAVCR2	PDCD1	TIGIT	TNFRSF9	ICOS	TLR4
CD27	LAG3	CD40	KIR2DL1	EDNRB		

Table S2. Primer sequences for genes.

Name	Sequence (5'→3')
B2M-F	GGAGGCTATCCAGCGTACTCCA
B2M-R	CGGATGGATGAAACCCAGACACA
HLA-B-F	GCCGCGAGTCCGAGGAC
HLA-B-R	CGCAGGTTCCGCAGG
HLA-C-F	CCATGAGGTATTTGTGGACCG
HLA-C-R	TCTCGGACTCTCGTCGTCG
HLA-DMA-F	ACTCACGAAATTGACCGCTACAC
HLA-DMA-R	CGCCACACAGCACATTCTCC
HLA-DMB-F	ACATACCAGACCCTCTCCCATTTAG
HLA-DMB-R	CAGGTGTCCAGTCCCGAAGG
HLA-DOA-F	CCTACGGACCCGCCTTCTA
HLA-DOA-R	GGCCTCGCTTTTCTTCAGG
HLA-DQA1-F	TGGGCAGTCAGTCACAGAAGG
HLA-DQA1-R	TCATCAGCAGAAGGGAGGAAGG
HLA-DPB1-F	CAGCACCACAACCTGCTTG
HLA-DPB1-R	CCATTCAGGAACCATCGGACT
HLA-DRA-F	AGTCCCTGTGCTAGGATTTTTCA
HLA-DRA-R	ACATAAACTCGCCTGATTGGTC
HLA-DRB-F	AGCGGCGAGTCCAACCTAAG
HLA-DRB-R	TTCAATGCTGCCTGGATAGAAACC
HLA-E-F	TTCCGAGTGAATCTGCGGAC
HLA-E-R	GTCGTAGGCGAACTGTTCATAC
KCNA6-F	TGCCTGTGCCCGTCATCG
KCNA6-R	TTGCCAAGTCCGTTGTCAGTTG
PIK3R3-F	GGATGCTATGCTTGCTCTGTGG
PIK3R3-R	CAAGGATGTCTGCTGGTAATGGAG
RGS5-F	CTTGAGTTCTGGATTGCCTGTGAG
RGS5-R	TTGTGATGTCCTTAGTGAAGTGGTC
TAP1-F	CTGGGGAAGTCACCCTACC
TAP1-R	CAGAGGCTCCCGAGTTTGTG
TAP2-F	TGGACGCGGCTTTACTGTG
TAP2-R	GCAGCCCTCTTAGCTTTAGCA
TAPBP-F	CCTGGAGGTAGCAGGTCTTTC
TAPBP-R	ATCCTTGCAGGTGGACAGGTA
TARDBP-F	GGTAACCGAAGATGAGAACGATGAG
TARDBP-R	CACTGGATTCCTGTAGCGAAGC
TOP2A-F	TCCGCCCAGACACCTACATTG
TOP2A-R	TGTCCGCAGCATTAACTAGAATCTC

β-actin-F	ATCACCATTGGCAATGAGCG
β-actin-R	TTGAAGGTAGTTTCGTGGAT

Table S3. Details for 8 LGG-related Hub-MHCsig.

Antibody	No.	Manufacturer
B2M	ab218230	Abcam
HLA-B	ab193415	Abcam
HLA-DOA	ab224668	Abcam
HLA-DPB1	ab224668	Abcam
HLA-DRA	ab92511	Abcam
HLA-E	abb300553	Abcam
TAP1	ab314745	Abcam
TAP2	ab307282	Abcam

Table S4. List of 21 MHC-related genes.

B2M	HLA-A	HLA-B	HLA-C	HLA-DMA	HLA-DMB	HLA-DOA
HLA-DOB	HLA-DPA1	HLA-DPB1	HLA-DQA1	HLA-DQA2	HLA-DQB1	HLA-DRA
HLA-DRB1	HLA-E	HLA-F	HLA-G	TAP1	TAP2	TAPBP

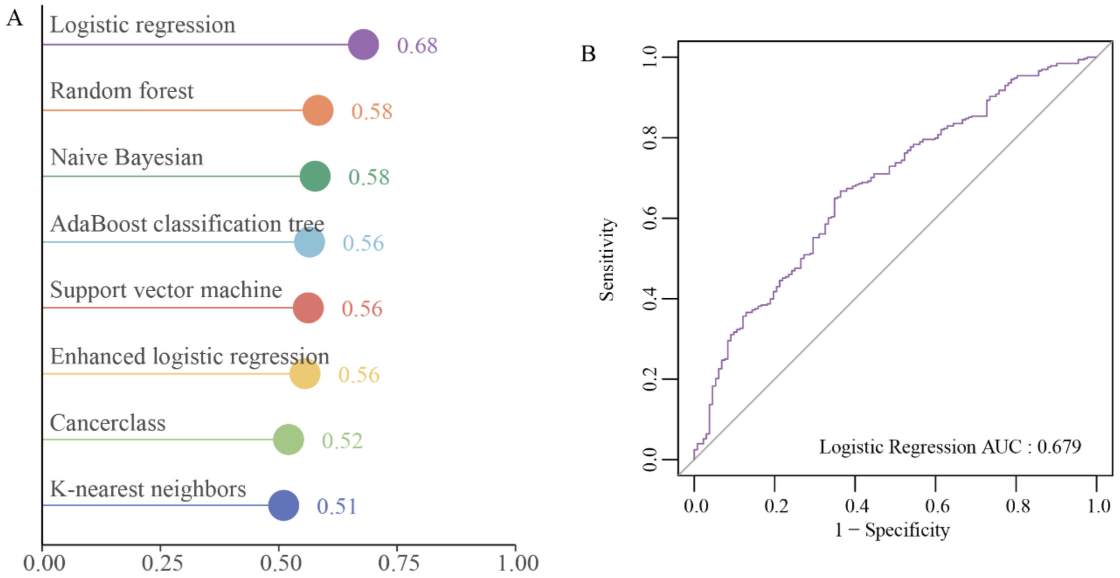

Figure S1. Prediction of immunotherapy outcomes using MHCsig. A. The performance of the prediction model constructed by 8 machine learning algorithms in the validation cohort. B. The receiver operating characteristic plot of the logistic regression model (MHCsig model) in the validation cohort.

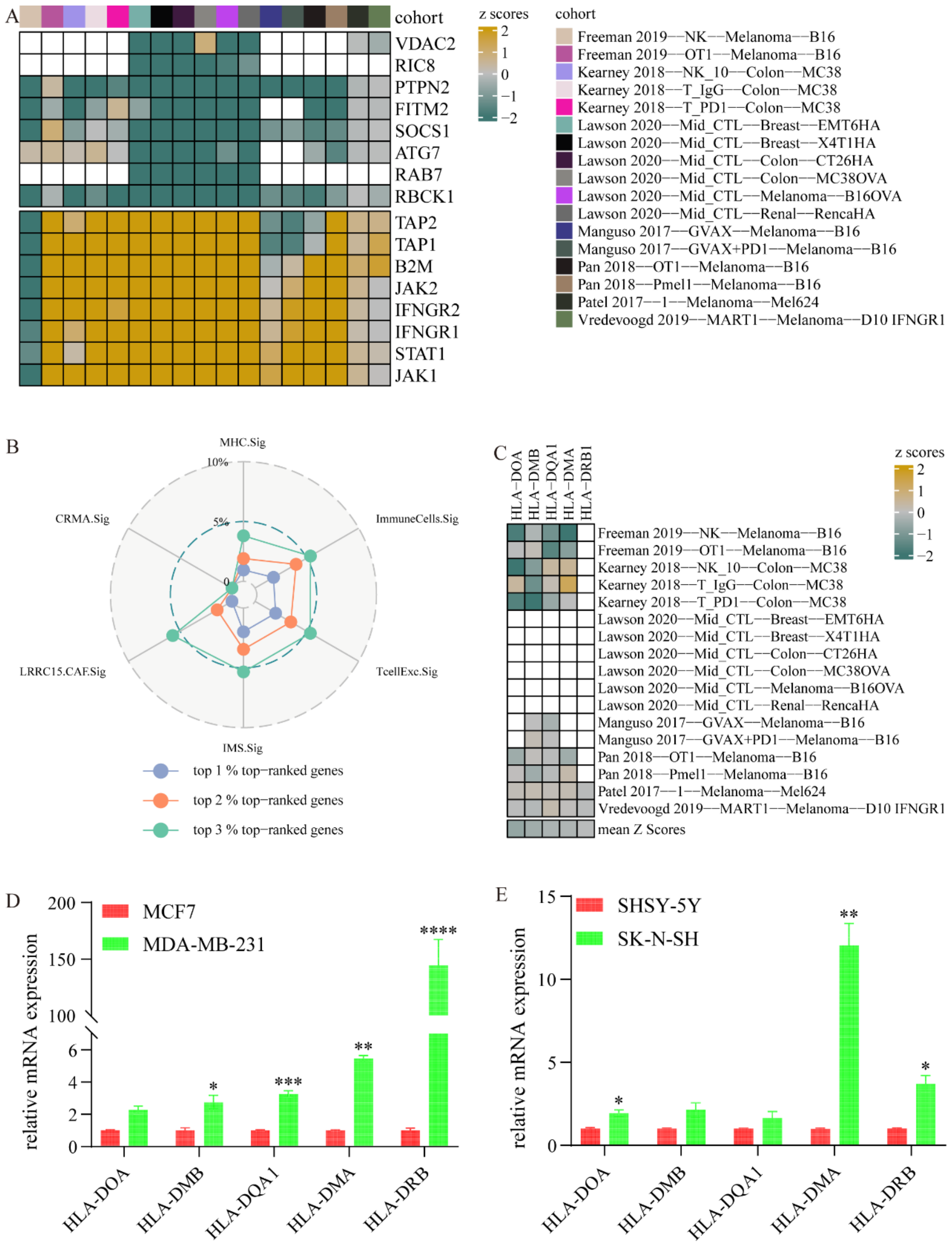

Figure S2. Screening of the potential therapeutic targets screened from MHCsig. A. Gene ranking based on knockdown effects on antitumor immunity in 17 CRISPR/Cas9 datasets. B. Radar plot comparing the percentage of top-ranked genes among MHCsig and other 5 immune-resistant signatures. C. Heatmap depicting z scores of 5 MHCsig in the 6% top-ranked genes in 17 CRISPR/Cas9 datasets. D.

Relative expression of 5 MHCsig in the breast cancer cells. E. Relative expression of 5 MHCsig in the neuroblastoma cells.

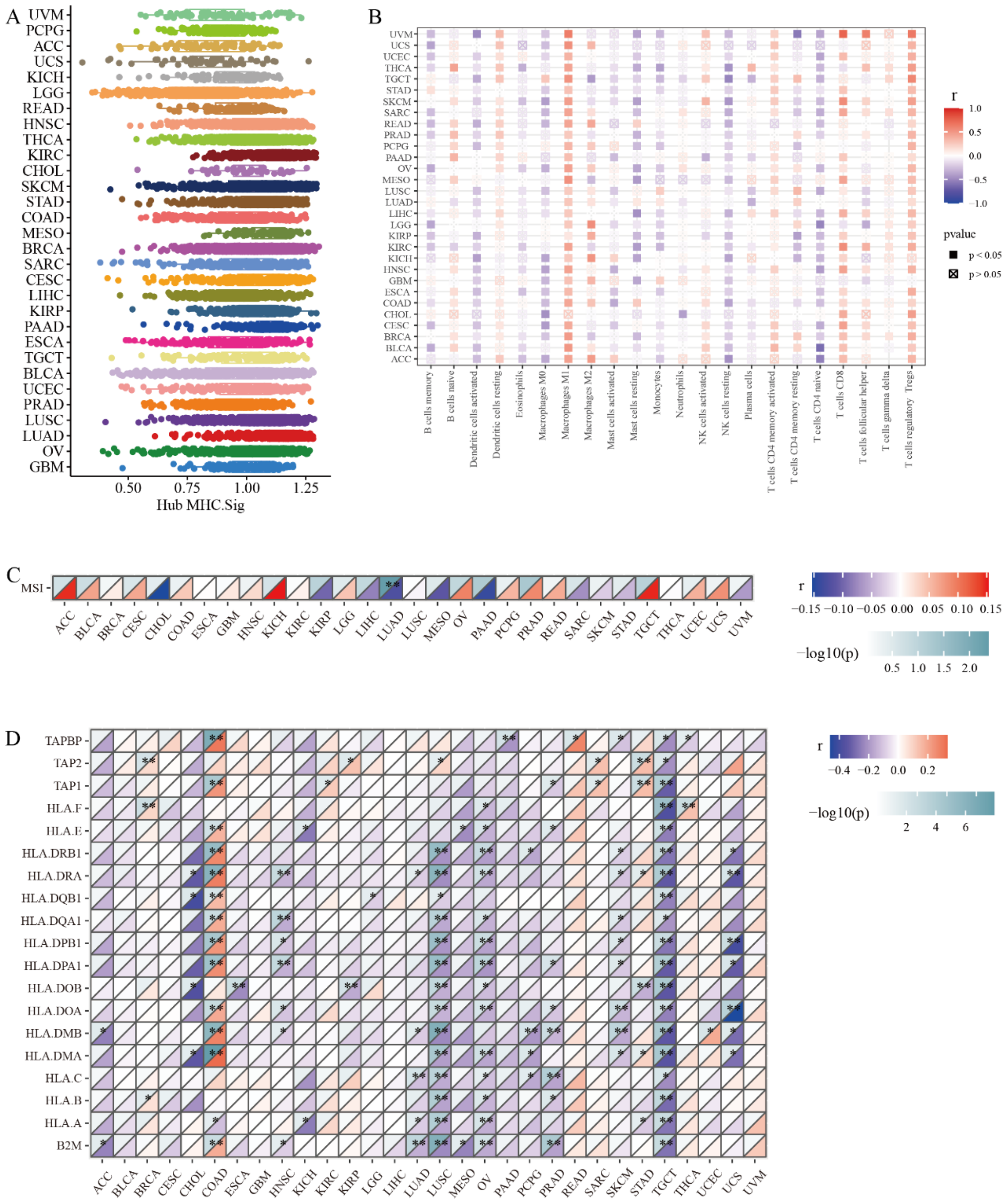

Figure S3. Landscape of Hub-MHCsig in pan-cancer. A. The scores of Hub-MHCsig in 30 cancers. B. The correlation between the scores of Hub-MHCsig and the abundance of immune cell infiltration. C. The correlation between the scores of Hub-MHCsig and microsatellite instability. D. The correlation between the 19 Hub-MHCsig and microsatellite instability.

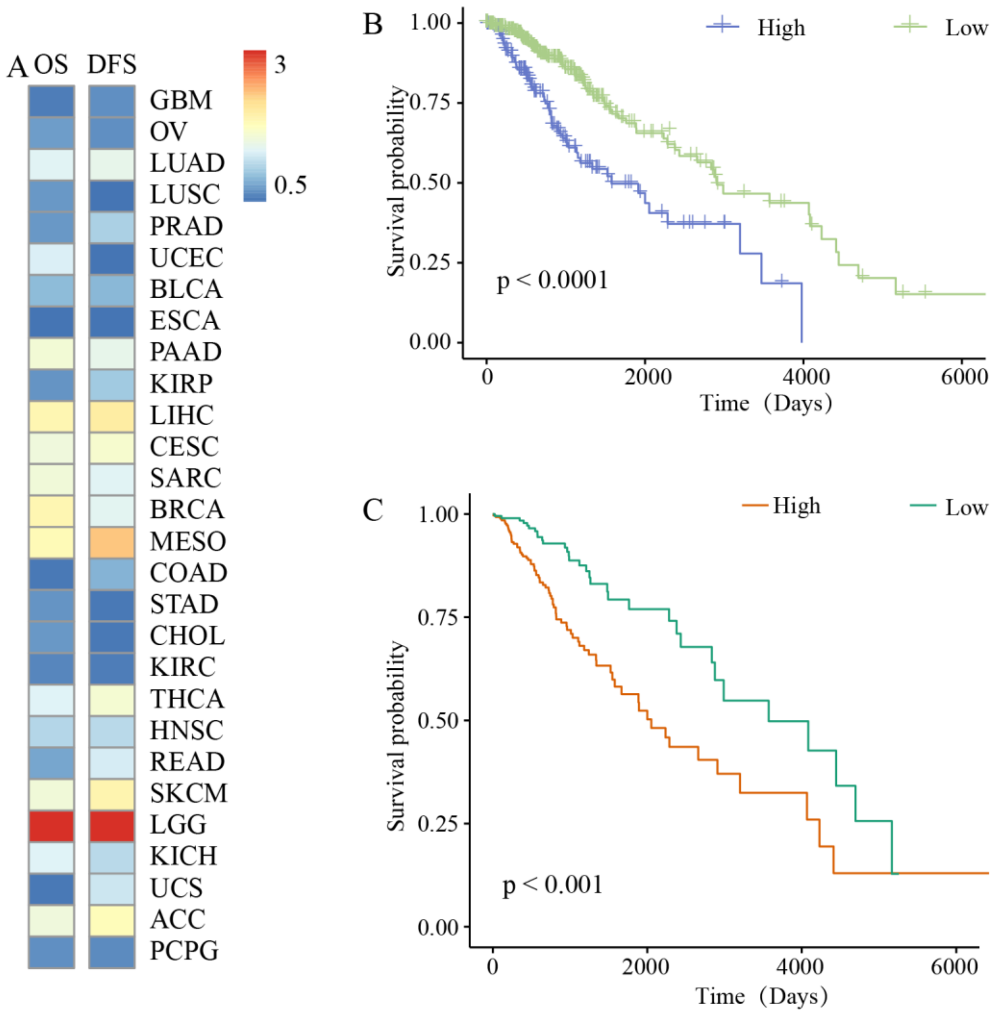

Figure S4. Survival analysis of Hub-MHCsig in pan-cancer. A. Overall survival and disease-special survival analysis of Hub-MHCsig in pan-cancer. B. Overall survival analysis of Hub-MHCsig in low-grade glioma. C. Disease-special survival analysis of Hub-MHCsig in low-grade glioma.

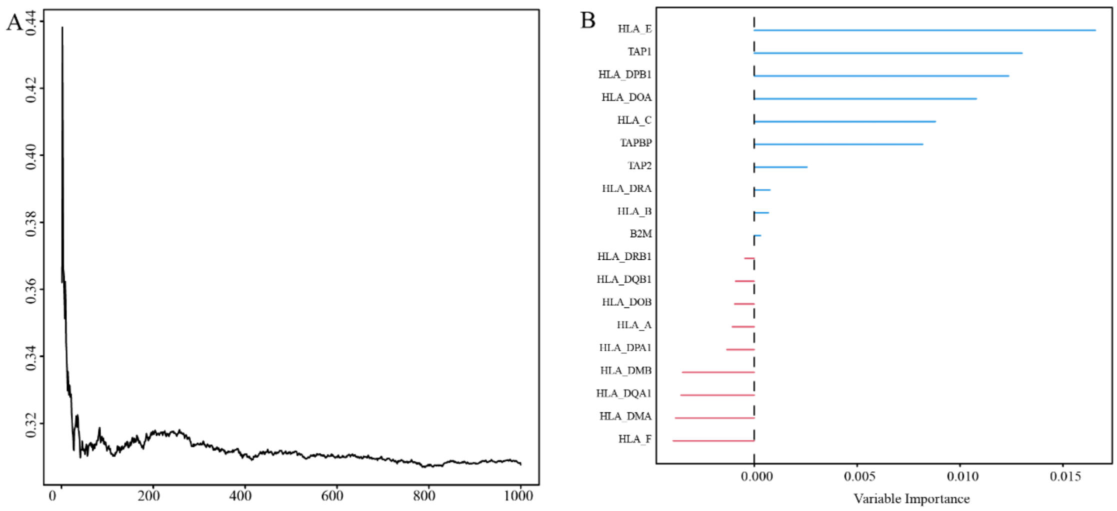

Figure S5. Screening of LGG-related Hub-MHCsig. A. Error rates of Hub-MHCsig in random forest model. B. Importance ranking plot of Hub-MHCsig in the random forest model.

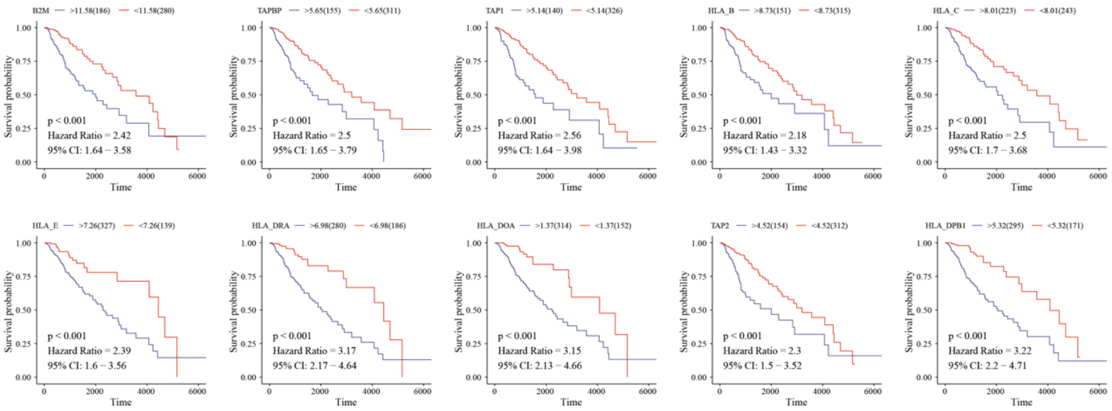

Figure S6. Disease-special survival analysis of 10 LGG-related Hub-MHCsig in LGG.

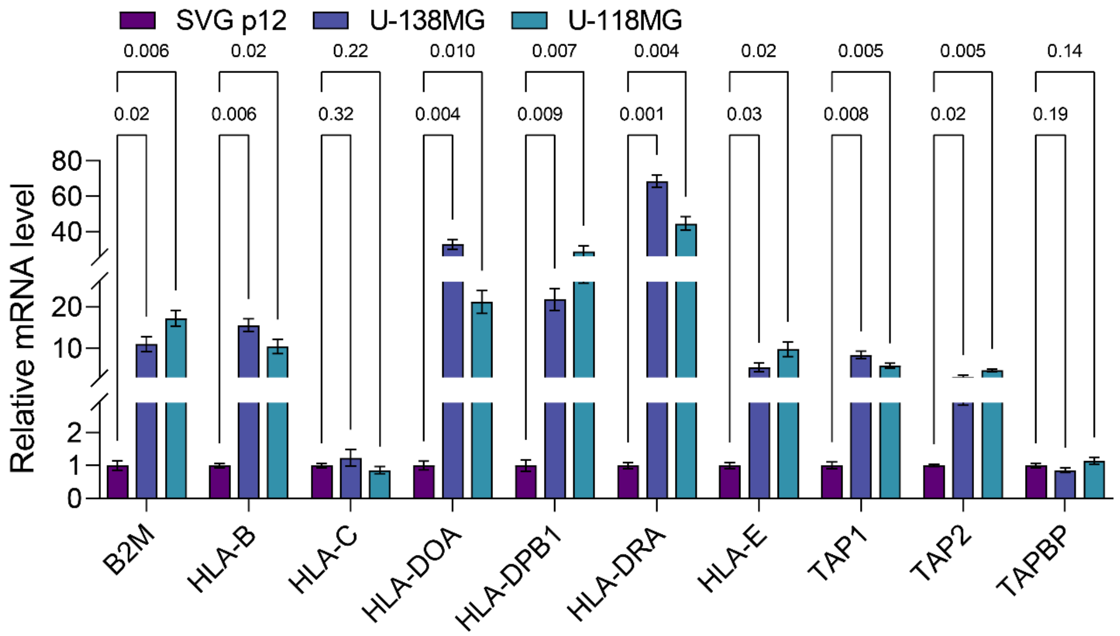

Figure S7. The mRNA expression of 10 LGG-related Hub-MHCsig in cell lines.

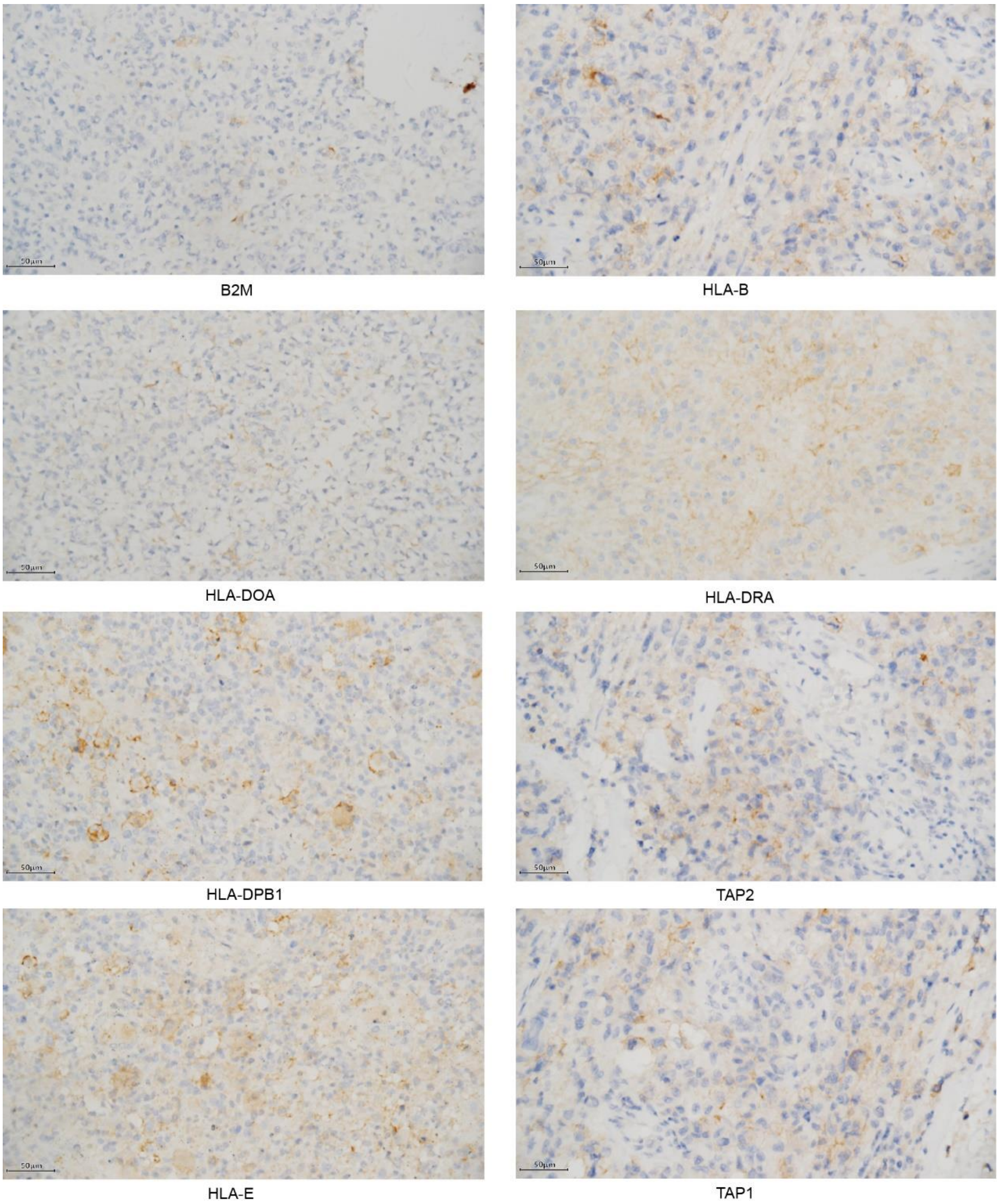

Figure S8. The expression of 8 LGG-related Hub-MHCsig in LGG patients from our clinical data.

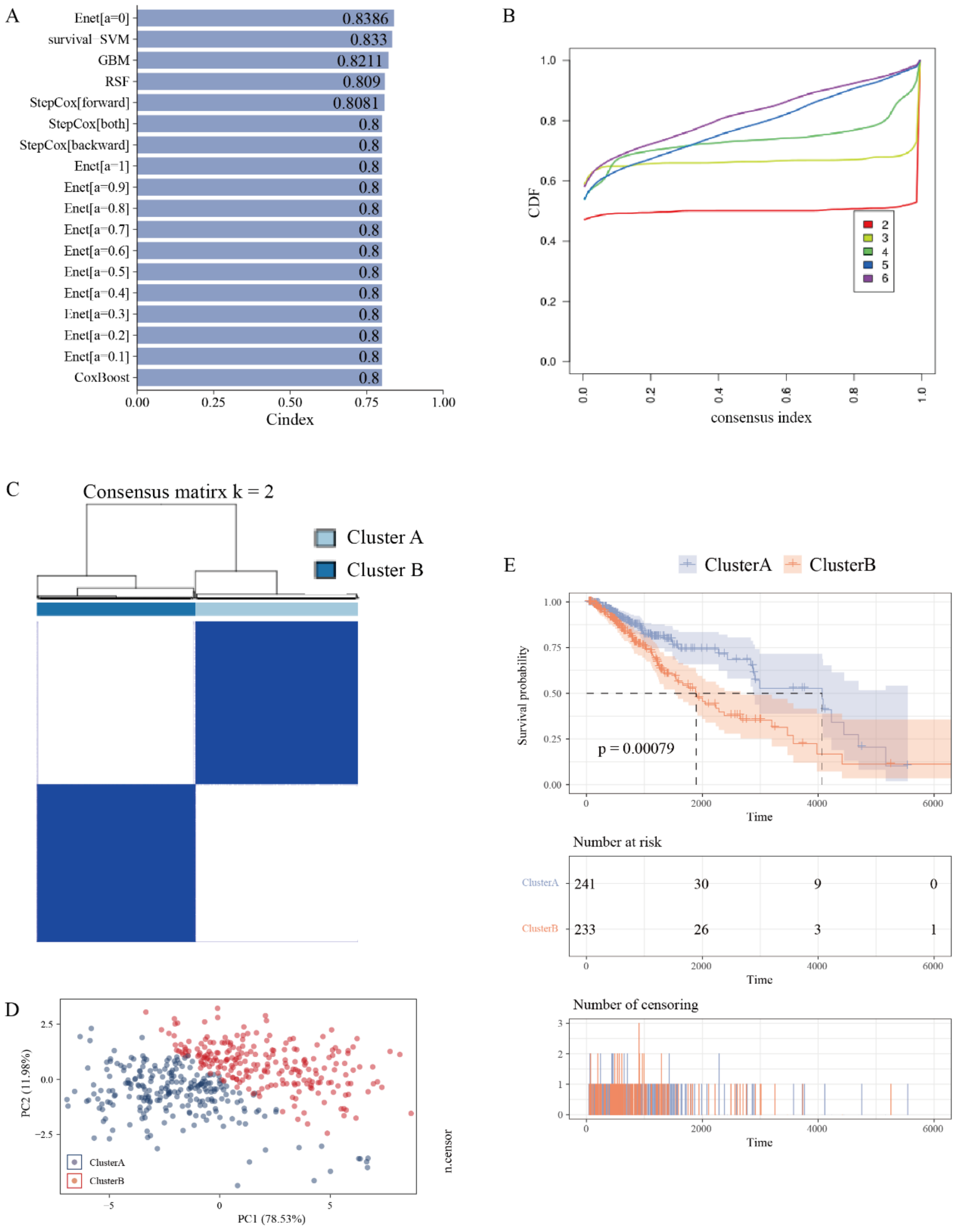

Figure S9. Construction of the prognostic model in LGG. A. 10 LGG-related Hub-MHCsig were used in the TCGA-BLCA dataset to construct a model for predicting patient prognosis through different machine learning algorithms. B. Cumulative distribution function. C. Two consensus clusters in the TCGA-BLCA dataset. D. Principal co-ordinates analysis. E. Survival analysis.

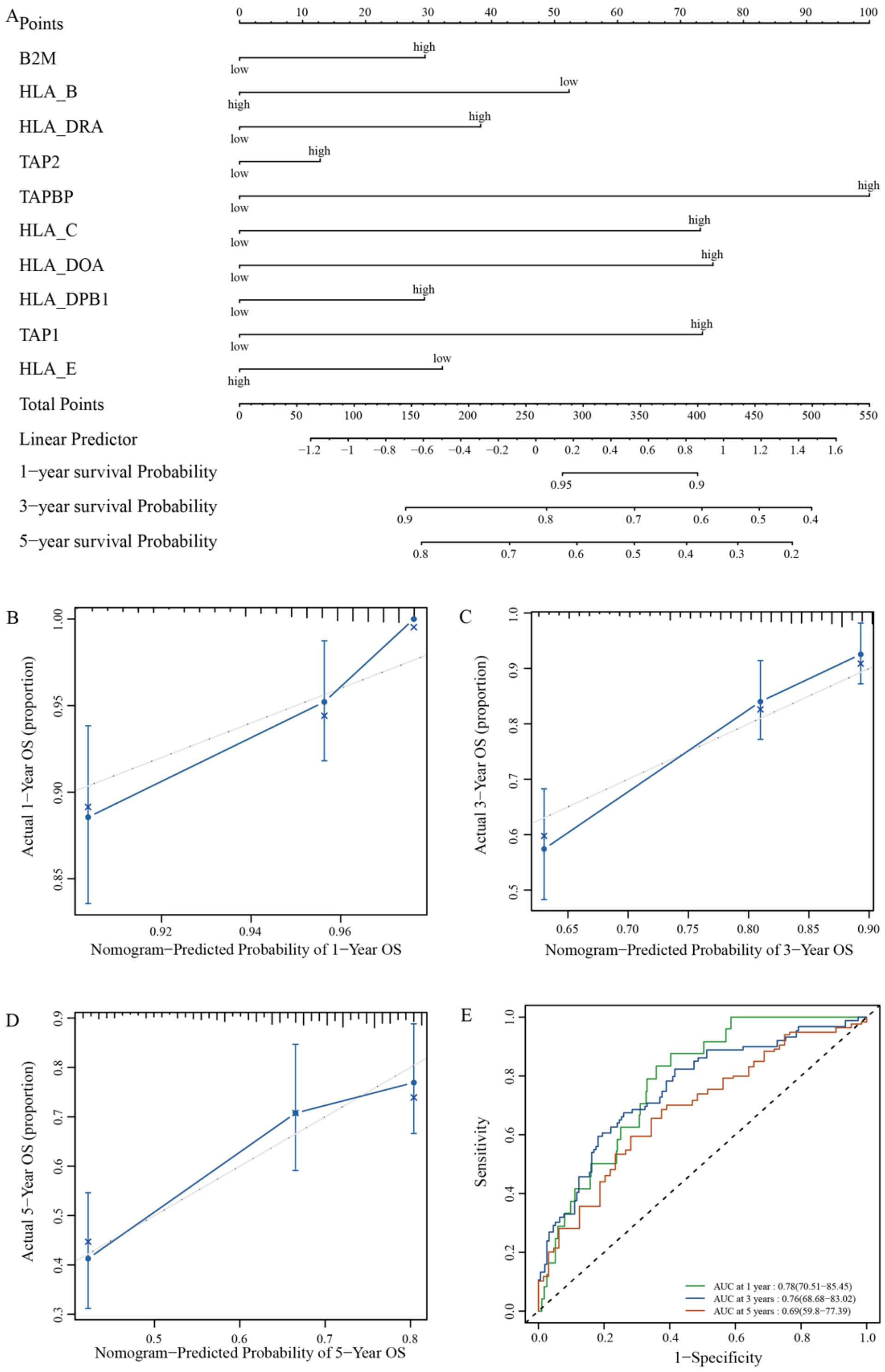

Figure S10. Nomogram contribution. A. Cox regression model nomogram of 10 LGG-related Hub-MHCsig. B. The 1-year calibration curve. C. The 3-year calibration curve. D. The 5-year calibration curve. E. The time-dependent ROC curves.

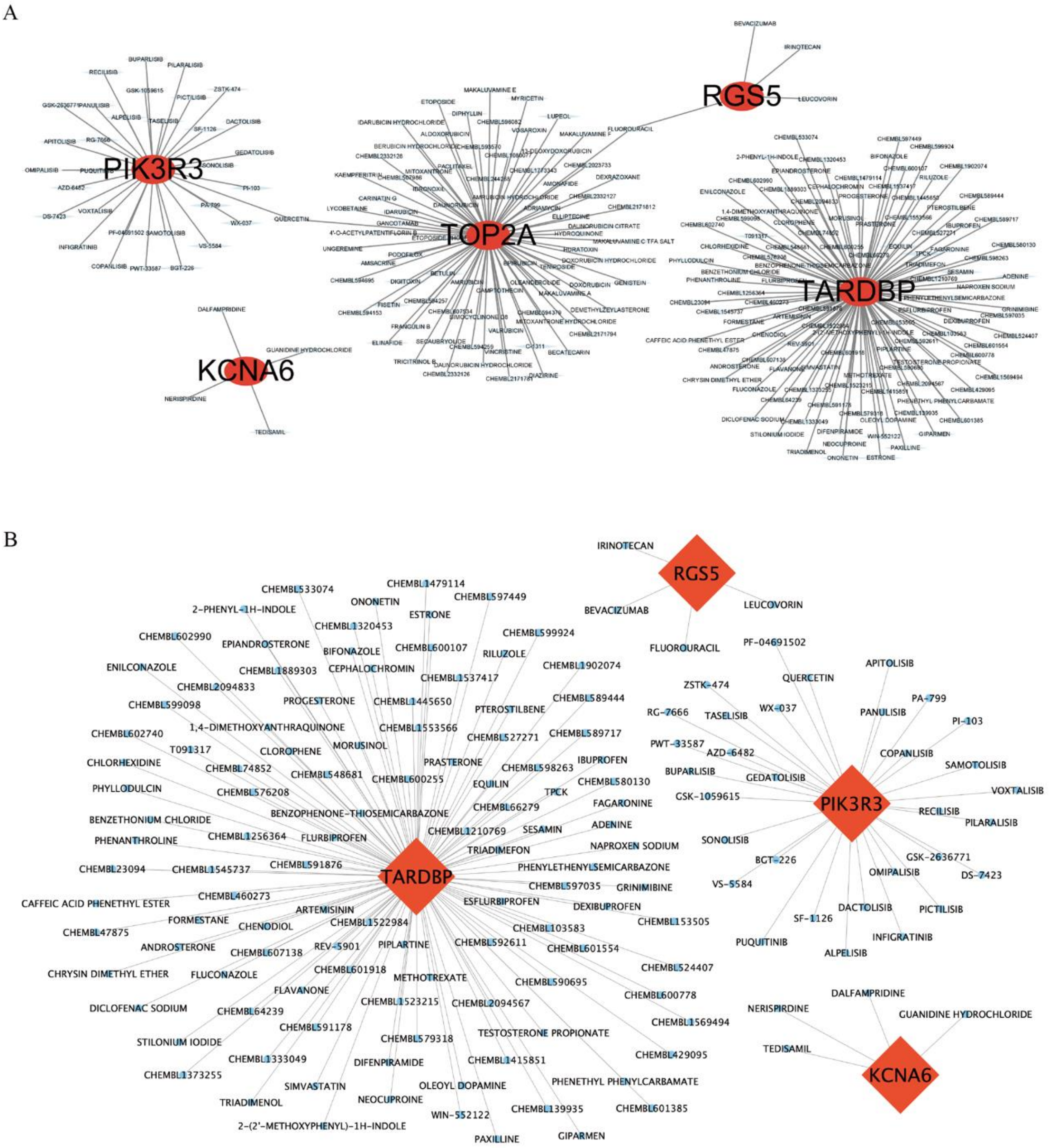

Figure S11. LGG-related Hub-MHCsig-drug networks. A. ClusterA. B. ClusterB.

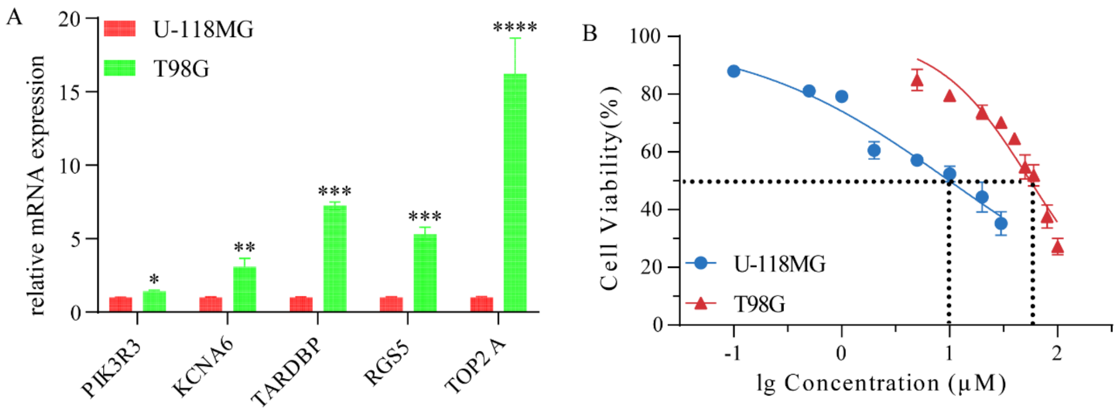

Figure S12. 5 LGG-related Hub-MHCsig in the LGG cells. A. Relative expression of 5 LGG-related Hub-MHCsig in the LGG cells. B. Half maximal inhibitory concentration of Adriamycin in the LGG cells.

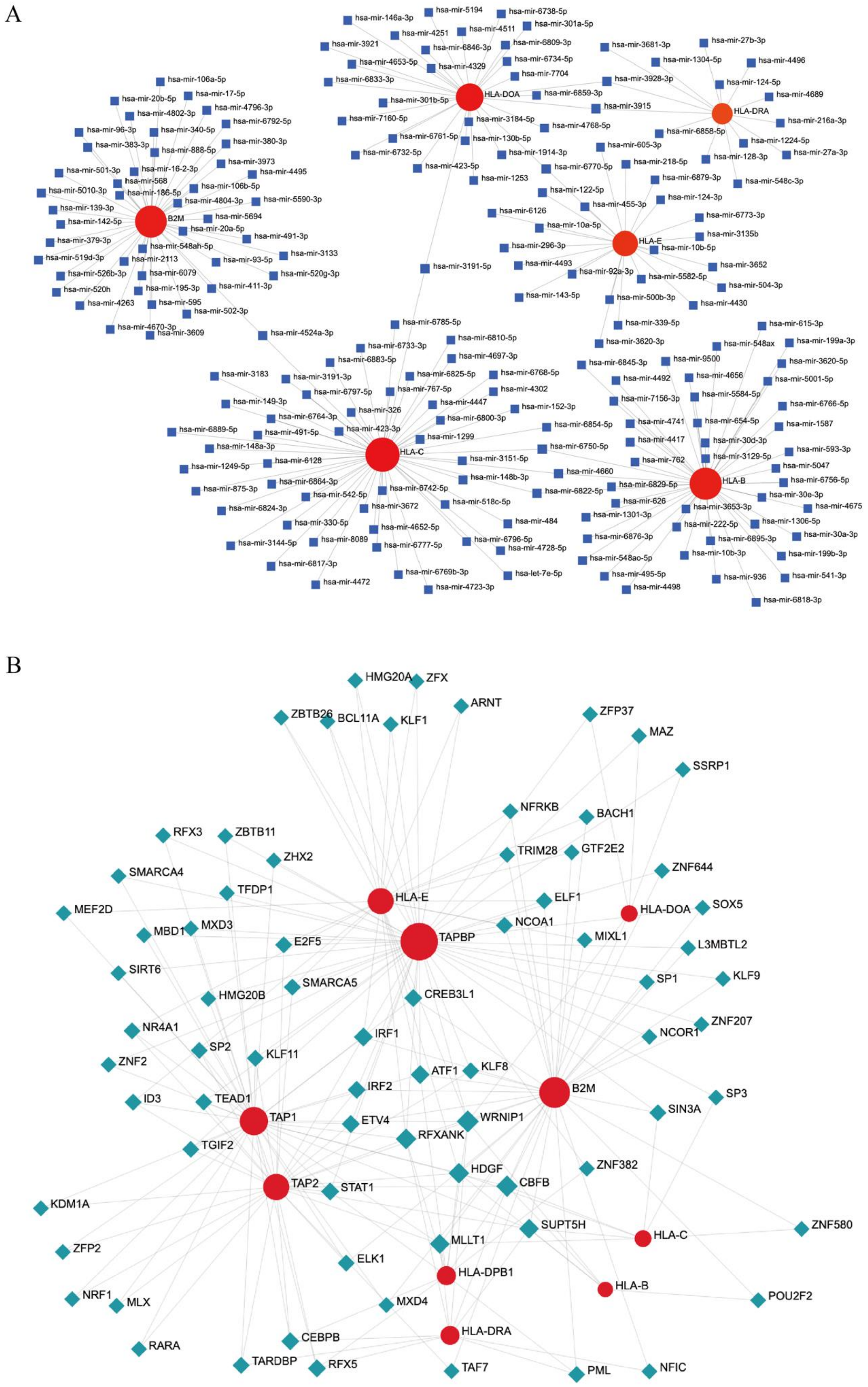
A
B2M
HLA-DOA
HLA-DRA
HLA-E
HLA-C
HLA-B
hsa-mir-106a-5p
hsa-mir-20b-5p
hsa-mir-17-5p
hsa-mir-4802-3p
hsa-mir-4796-3p
hsa-mir-96-3p
hsa-mir-340-5p
hsa-mir-6792-5p
hsa-mir-383-3p
hsa-mir-888-5p
hsa-mir-380-3p
hsa-mir-3973
hsa-mir-501-3p
hsa-mir-16-2-3p
hsa-mir-4495
hsa-mir-568
hsa-mir-106b-5p
hsa-mir-5010-3p
hsa-mir-186-5p
hsa-mir-4804-3p
hsa-mir-5590-3p
hsa-mir-139-3p
hsa-mir-142-5p
hsa-mir-5694
hsa-mir-20a-5p
hsa-mir-379-3p
hsa-mir-491-3p
hsa-mir-548ah-5p
hsa-mir-519d-3p
hsa-mir-2113
hsa-mir-93-5p
hsa-mir-3133
hsa-mir-526b-3p
hsa-mir-6079
hsa-mir-520g-3p
hsa-mir-520h
hsa-mir-195-3p
hsa-mir-411-3p
hsa-mir-4263
hsa-mir-595
hsa-mir-502-3p
hsa-mir-4670-3p
hsa-mir-3609
hsa-mir-4524a-3p
hsa-mir-146a-3p
hsa-mir-5194
hsa-mir-6738-5p
hsa-mir-4251
hsa-mir-4511
hsa-mir-301a-5p
hsa-mir-3921
hsa-mir-6846-3p
hsa-mir-6809-3p
hsa-mir-4653-5p
hsa-mir-4329
hsa-mir-6734-5p
hsa-mir-7704
hsa-mir-6833-3p
hsa-mir-6859-3p
hsa-mir-301b-5p
hsa-mir-7160-5p
hsa-mir-3184-5p
hsa-mir-4768-5p
hsa-mir-6761-5p
hsa-mir-130b-5p
hsa-mir-6732-5p
hsa-mir-1914-3p
hsa-mir-423-5p
hsa-mir-1253
hsa-mir-3191-5p
hsa-mir-3681-3p
hsa-mir-27b-3p
hsa-mir-1304-5p
hsa-mir-4496
hsa-mir-3928-3p
hsa-mir-124-5p
hsa-mir-4689
hsa-mir-3915
hsa-mir-216a-3p
hsa-mir-6858-5p
hsa-mir-1224-5p
hsa-mir-605-3p
hsa-mir-128-3p
hsa-mir-27a-3p
hsa-mir-6770-5p
hsa-mir-218-5p
hsa-mir-548c-3p
hsa-mir-6879-3p
hsa-mir-122-5p
hsa-mir-124-3p
hsa-mir-455-3p
hsa-mir-6126
hsa-mir-6773-3p
hsa-mir-10a-5p
hsa-mir-3135b
hsa-mir-296-3p
hsa-mir-10b-5p
hsa-mir-4493
hsa-mir-3652
hsa-mir-92a-3p
hsa-mir-5582-5p
hsa-mir-504-3p
hsa-mir-143-5p
hsa-mir-500b-3p
hsa-mir-4430
hsa-mir-6785-5p
hsa-mir-339-5p
hsa-mir-615-3p
hsa-mir-6810-5p
hsa-mir-3620-3p
hsa-mir-548ax
hsa-mir-199a-3p
hsa-mir-6733-3p
hsa-mir-4697-3p
hsa-mir-9500
hsa-mir-6883-5p
hsa-mir-6845-3p
hsa-mir-3620-5p
hsa-mir-3183
hsa-mir-3191-3p
hsa-mir-6825-5p
hsa-mir-6768-5p
hsa-mir-4492
hsa-mir-4656
hsa-mir-5001-5p
hsa-mir-6797-5p
hsa-mir-767-5p
hsa-mir-4302
hsa-mir-7156-3p
hsa-mir-5584-5p
hsa-mir-149-3p
hsa-mir-4447
hsa-mir-152-3p
hsa-mir-6766-5p
hsa-mir-326
hsa-mir-6764-3p
hsa-mir-6800-3p
hsa-mir-6889-5p
hsa-mir-491-5p
hsa-mir-423-3p
hsa-mir-6854-5p
hsa-mir-4741
hsa-mir-654-5p
hsa-mir-1587
hsa-mir-148a-3p
hsa-mir-1299
hsa-mir-4417
hsa-mir-30d-3p
hsa-mir-6128
hsa-mir-6750-5p
hsa-mir-593-3p
hsa-mir-1249-5p
hsa-mir-3151-5p
hsa-mir-762
hsa-mir-3129-5p
hsa-mir-5047
hsa-mir-4660
hsa-mir-148b-3p
hsa-mir-6756-5p
hsa-mir-875-3p
hsa-mir-6864-3p
hsa-mir-6742-5p
hsa-mir-6829-5p
hsa-mir-6822-5p
hsa-mir-542-5p
hsa-mir-30e-3p
hsa-mir-626
hsa-mir-518c-5p
hsa-mir-4675
hsa-mir-6824-3p
hsa-mir-3672
hsa-mir-3653-3p
hsa-mir-1301-3p
hsa-mir-484
hsa-mir-1306-5p
hsa-mir-330-5p
hsa-mir-4652-5p
hsa-mir-222-5p
hsa-mir-3144-5p
hsa-mir-8089
hsa-mir-6796-5p
hsa-mir-6876-3p
hsa-mir-6895-3p
hsa-mir-30a-3p
hsa-mir-6777-5p
hsa-mir-4728-5p
hsa-mir-6817-3p
hsa-mir-548ao-5p
hsa-mir-10b-3p
hsa-mir-199b-3p
hsa-mir-6769b-3p
hsa-let-7e-5p
hsa-mir-495-5p
hsa-mir-936
hsa-mir-541-3p
hsa-mir-4472
hsa-mir-4723-3p
hsa-mir-4498
hsa-mir-6818-3p
B
HMG20A
ZFX
ARNT
ZBTB26
BCL11A
KLF1
ZFP37
MAZ
SSRP1
NFRKB
BACH1
RFX3
ZBTB11
ZHX2
TRIM28
GTF2E2
SMARCA4
ZNF644
TFDP1
HLA-E
ELF1
SOX5
MEF2D
HLA-DOA
MXD3
NCOA1
MBD1
TAPBP
E2F5
MIXL1
L3MBTL2
SIRT6
SP1
KLF9
SMARCA5
HMG20B
CREB3L1
NR4A1
ZNF207
NCOR1
IRF1
SP2
KLF11
ZNF2
KLF8
ATF1
IRF2
B2M
SP3
ID3
TEAD1
SIN3A
TAP1
WRNIP1
ETV4
RFXANK
TGIF2
ZNF382
HDGF
CBFB
TAP2
STAT1
KDM1A
SUPT5H
ZNF580
HLA-C
MLLT1
ZFP2
ELK1
HLA-DPB1
HLA-B
NRF1
MLX
MXD4
POU2F2
HLA-DRA
CEBPB
RARA
TARDBP
RFX5
TAF7
PML
NFIC

Figure S13. Regulatory networks of 10 LGG-related Hub-MHCsig. A. LGG-related Hub-MHCsig-miRNA network. B. LGG-related Hub-MHCsig-transcription factor network.
